# Thioridazine Induces Major Changes in Global Gene Expression and Cell Wall Composition in Methicillin-Resistant *Staphylococcus aureus* USA300

**DOI:** 10.1371/journal.pone.0064518

**Published:** 2013-05-17

**Authors:** Mette Thorsing, Janne K. Klitgaard, Magda L. Atilano, Marianne N. Skov, Hans Jørn Kolmos, Sérgio R. Filipe, Birgitte H. Kallipolitis

**Affiliations:** 1 Department of Biochemistry and Molecular Biology, University of Southern Denmark, Odense, Denmark; 2 Department of Clinical Research, Research Unit of Clinical Microbiology, University of Southern Denmark, Odense, Denmark; 3 Laboratory of Bacterial Cell Surfaces and Pathogenesis, Instituto de Tecnologia Química e Biológica, Universidade Nova de Lisboa, Oeiras, Portugal; National Institutes of Health, United States of America

## Abstract

Subinhibitory concentrations of the neuroleptic drug thioridazine (TDZ) are well-known to enhance the killing of methicillin-resistant *Staphylococcus aureus* (MRSA) by β-lactam antibiotics, however, the mechanism underlying the synergy between TDZ and β-lactams is not fully understood. In the present study, we have examined the effect of a subinhibitory concentration of TDZ on antimicrobial resistance, the global transcriptome, and the cell wall composition of MRSA USA300. We show that TDZ is able to sensitize the bacteria to several classes of antimicrobials targeting the late stages of peptidoglycan (PGN) synthesis. Furthermore, our microarray analysis demonstrates that TDZ modulates the expression of genes encoding membrane and surface proteins, transporters, and enzymes involved in amino acid biosynthesis. Interestingly, resemblance between the transcriptional profile of TDZ treatment and the transcriptomic response of *S. aureus* to known inhibitors of cell wall synthesis suggests that TDZ disturbs PGN biosynthesis at a stage that precedes transpeptidation by penicillin-binding proteins (PBPs). In support of this notion, dramatic changes in the muropeptide profile of USA300 were observed following growth in the presence of TDZ, indicating that TDZ can interfere with the formation of the pentaglycine branches. Strikingly, the addition of glycine to the growth medium relieved the effect of TDZ on the muropeptide profile. Furthermore, exogenous glycine offered a modest protective effect against TDZ-induced β-lactam sensitivity. We propose that TDZ exposure leads to a shortage of intracellular amino acids, including glycine, which is required for the production of normal PGN precursors with pentaglycine branches, the correct substrate of *S. aureus* PBPs. Collectively, this work demonstrates that TDZ has a major impact on the cell wall biosynthesis pathway in *S. aureus* and provides new insights into how MRSA may be sensitized towards β-lactam antibiotics.

## Introduction

Infections caused by methicillin-resistant *Staphylococcus aureus* (MRSA) were once mainly associated with risk factors such as hospitalization; however, within the last decade community-acquired MRSA (CA-MRSA) clones have rapidly disseminated throughout many industrialized regions of the world, infecting previously healthy individuals [1]. Most often these clonal lineages give rise to skin and soft tissue infections, but CA-MRSA have also been associated with severe conditions like necrotizing pneumonia and sepsis [1], [2]. In the United States and Canada, strains of the hypervirulent USA300 clone are the predominant cause of CA-MRSA infections [1], [3]; moreover, USA300 has subsequently spread to multiple countries outside of North America raising the possibility of an international epidemic [4].

In response to the rapid development of resistance, new treatment strategies for MRSA infections are considered. The phenothiazines, a class of neuroleptic drugs, are well known to sensitize MRSA to β-lactam antibiotics [5]–[7], which was originally contributed to their inhibitory effect on multidrug efflux pumps [5], [8]. Phenothiazines interact strongly with and alter the biophysical properties of lipid bilayers [9]–[11], and have been shown to interfere with various membrane-based processes including respiration [12], , ion fluxes [14]–[16], and glycine uptake [17], [18]. Many of these effects have been shown to apply to both prokaryotes and eukaryotes hinting at a more general mode of action. Previously, we have shown that the phenothiazine derivate thioridazine (TDZ) (Figure S1) reduces the level of the MRSA resistance determinant PBP2a [6] and affects the expression of several genes involved in cell wall biosynthesis [19]. Furthermore, we recently demonstrated that TDZ increases the β-lactam sensitivity of methicillin-sensitive *S. aureus* (MSSA), showing that additional factors besides PBP2a must be involved in the mechanism underlying the synergy between TDZ and β-lactam antibiotics against *S. aureus* [7]. In continuation hereof, this study presents a global transcriptional analysis of the response of USA300 to TDZ and the effects of TDZ on the muropeptide composition of the peptidoglycan (PGN) produced by *S. aureus* USA300. Collectively, the results of these analyses suggest that TDZ interferes with amino acid metabolism and, consequently, the biosynthesis of PGN, and provide a broader understanding of how TDZ sensitizes MRSA strains to β-lactam antibiotics.

## Materials and Methods

### Bacterial strains and growth conditions

MRSA strain USA300 FPR3757 (ATCC BAA-1556) was used throughout these studies. Methicillin-sensitive *S. aureus* strain Newman (ATCC 25904) was used for membrane preparations. Unless otherwise mentioned, a colony was picked from a Mueller-Hinton (MH, Merck) agar plate and grown in MH broth for 18–20 hours. The overnight culture was diluted in brain heart infusion broth (BHI, Oxoid) to OD_600_ 0.02 and grown at 37°C with shaking.

### MIC determination

Minimal inhibitory concentrations (MIC) for dicloxacillin (DCX, Bristol-Myers Squibb) and thioridazine hydrochloride (TDZ, Sigma-Aldrich) were determined by the broth microdilution method according to [20] in BHI broth. The MIC for lysostaphin (Sigma-Aldrich) was determined in BHI and BHI supplemented with 16 µg/mL TDZ. 100 µl of medium containing twofold serial dilutions of the drugs were inoculated with ∼5×10^4^ bacteria and OD_450_ values were determined after 24 hours of growth at 37°C using a 96-well plate reader (VICTOR^3^, PerkinElmer). MIC values were determined in quadruplicates.

### Growth and time-kill assays

To find the suitable drug concentrations for the time-kill assays, USA300 was grown to early exponential phase and treated with two-fold serial dilutions of TDZ, DCX, phosphomycin disodium salt (FOS, Sigma-Aldrich), D-cycloserine (CYC, Sigma-Aldrich), bacitracin from *Bacillus licheniformis* (BAC, Sigma-Aldrich), or vancomycin hydrochloride (VAN, Sigma-Aldrich). Growth was monitored by OD_600_ measurements and the highest concentration of the drugs allowing the bacteria to reach the same stationary OD_600_ value as the untreated culture was chosen.

Viability of USA300 during treatment with TDZ alone or in combination with cell wall active antibiotics was assessed as previously described [6]. Briefly, a culture was grown to OD_600_ 0.20 (early exponential growth phase), split, and treated with TDZ and antibiotics alone or in combination. An untreated control was included. In experiments examining the effect of exogenous glycine, BHI was supplemented with glycine (sterile 2 M stock solution) to a final concentration of 10 mM simultaneously with the addition of antimicrobial drugs. Viability was monitored by OD_600_ readings and CFU/mL determinations by plating 10-fold serial dilutions of the cultures on MH agar plates. The viability assays were repeated twice with similar results.

### Population analysis

Cultures were grown as described for the time-kill assay in the absence and presence of 16 µg/mL TDZ. After 8 hours of treatment the susceptibility to DCX was determined by plating 10-fold serial dilutions onto control plates without antibiotics and onto plates containing a series of two-fold dilutions of DCX (0.1–1000 µg/mL). The plates were incubated at 37°C for 48 hours, and the colonies were counted. For each value the mean of ten determinations from two independent experiments is shown.

### Isolation of total cellular RNA

Triplicate overnight cultures of USA300 were prepared, diluted, and treated with TDZ and/or DCX as described for the time-kill assay. After 30 min of treatment, cultures were stabilized with 2 volumes RNAprotect Bacteria Reagent (QIAGEN) as instructed by the manufacturer. RNA was purified by a hot acid-phenol procedure [21] and treated with 20 U DNase I (Roche) for 30 min at 37°C. The purification procedure was repeated in order to remove the DNase. RNA concentration and quality was analyzed using a 2100 Bioanalyzer (Agilent), which confirmed the absence of genomic DNA.

### DNA microarray analysis

Purified RNA was sent to Roche NimbleGen (Madison, WI) for cDNA synthesis, labeling, hybridization, and quantification according to company protocols. A 12×135 K chip (110204_Saureus_FPR3757_JK_exp_HX12; Roche NimbleGen) was custom designed for *S. aureus* USA300 FPR3757 (NC_007793) covering 2563 of the 2605 ORFs in the genome and the three plasmids (NC_007790.1, NC_007791.1, and NC_007792.1). Each target was covered by ten 60-mer probes in five replicates. The complete microarray data set is available at the NCBI Gene Expression Omnibus (GEO) database under accession number GSE43759.

### Data analysis

Gene expression data files provided by NimbleGen contained the average signal intensity for the individual probes on each array. All statistical analyses were accomplished using R program language. Gene expression data was preprocessed using the BioConductor Oligo package based on the Robust Multichip Average (RMA) method [22]. To find differentially expressed genes, the Empirical Bayes method employed in the LIMMA (linear models for microarray analysis) package [23] was used to calculate a moderated t-statistic. P-values were calculated based on the moderated t-statistic and adjusted for multiple testing using the Benjamini-Hochberg correction. Genes with a fold change ≥2 and an adjusted *p*-value <0.05 were considered significantly differentially expressed.

### Verification of microarray data by RT-qPCR

cDNA templates were created from 150 ng RNA using the Maxima First Strand cDNA Synthesis Kit for RT-qPCR (Fermentas) according to the recommended procedure. Primer sets were designed using Primer3 [24] for *mecA, relA, saeR, sirA, saeP, cspB, coa, ctsR, sarA, oppB, SAUSA300_0986, ilvD, spsA, accB*, and *vraR* to generate amplicons of approximately 80 bp (Table S1). *gyrB* and *rpoA* served as reference genes. cDNA was amplified using the Maxima SYBR Green/ROX qPCR Master Mix (Fermentas) on an MX-3000P system (Agilent) with an initial incubation at 95°C for 10 min followed by 40 cycles of 15 sec at 95°C, 15 sec at 60°C, and 30 sec at 72°C. Absence of primer-dimers was confirmed by generation of a melting curve at the end of the program. Data was analyzed using “Relative Expression Software Tool - Multiple Condition Solver” [25], [26]. For statistical analyses, Students *t*-test was performed. The differences reported were statistically significant with at least 95% confidence.

### Transmission electron microscopy (TEM)

USA300 was grown to early exponential phase as described above. To ensure continued exponential growth, the culture was diluted 5 fold in fresh BHI containing TDZ (final concentration: 16 µg/mL), DCX (final concentration: 0.125 µg/mL), or TDZ and DCX in combination. An untreated control was included. The cultures were treated for 2.5 hours at 37°C with shaking after which the bacteria were collected by centrifugation and washed twice with PBS. Pellets were fixed overnight at room temperature in 2% glutaraldehyde in 0.04 M phosphate buffer (pH 7.4), washed once in 0.1 M phosphate buffer (pH 7.4), and resuspended in 15% BSA solution. Samples were incubated for 60 min at 20°C, centrifuged, and fixed overnight as described above, but at 4°C. Cell material was cut into appropriate pieces, washed three times in 0.1 M phosphate buffer, and stained with 1% OsO_4_ in 0.05 M phosphate buffer for 60 min at 4°C. Cells were dehydrated at 4°C with increasing concentrations of ethanol (50–99%) and embedded in epon. Ultra-thin sections (60 nm) were stained with 3% uranyl acetate for 14 min at 60°C, washed in water, stained with lead citrate (Leica Ultrastain 2) for 6 min at room temperature, and washed in 20 mM NaOH. Sections were viewed and photographed using a Philips TEM 208 microscope.

### Fluorescence microscopy

USA300 was grown in BHI broth for 7–8 generations to mid-exponential phase in the absence and presence of 16 µg/mL TDZ or 0.125 µg/mL DCX. Cells were stained with the cell wall dye Van-FL (1 µg/mL, Molecular Probes) and DNA dye Hoechst 33342 (1 µg/mL, Molecular Probes) and observed on a thin film of 1% agarose in PBS by fluorescence microscopy using a Zeiss Axio Observer.Z1 microscope equipped with a Photometrics CoolSNAP HQ2 camera (Roper Scientific) and Metamorph software (Meta Imaging series 7.5). Scale bars correspond to 1 µm. Membrane integrity was examined by staining cells with the LIVE/DEAD BacLight Bacterial Viability Kit (Molecular Probes) and imaging them using a Leica DMRE microscope equipped with a DC500 camera using IM50 software.

### PBP extraction and profiling

Membranes were prepared from exponential phase cultures of USA300 or Newman grown for 7–8 generations with or without 16 µg/mL TDZ. The cultures were chilled, harvested, and washed once in PBS. Pellets were resuspended in 50 mM phosphate buffer (pH 7.4), 10 mM MgCl_2_ containing Complete ULTRA™ EDTA-free protease inhibitors (Roche; 1 tablet per10 mL buffer) and cells were broken with glass beads in a FastPrep FP120 (Qbiogene). Glass beads were removed by low speed centrifugation and lysates were incubated with 100 µg/mL lysostaphin (Sigma-Aldrich), 10 µg/mL DNase I (Sigma-Aldrich), and 10 µg/mL RNase A (Sigma-Aldrich) for 1 hour at room temperature with gentle agitation. Cell debris was removed by centrifugation (30 min, 7000 x*g*, 4°C) and membranes were collected from the supernatant by ultracentrifugation (1 h, 100,000 x*g*, 4°C). Membranes were washed once in sample buffer (50 mM phosphate buffer (pH 7.4), 10 mM MgCl_2_, 20% glycerol) and protein content was quantified using the BCA Protein Assay Kit (Thermo Scientific Pierce).

Membranes (50 µg) were preincubated with TDZ or DCX in sample buffer for 15 min at 37°C and then labeled with 10 µM Bocilin-FL for 10 min at 37°C. Reactions were stopped by adding 5× SDS-PAGE loading buffer (500 mM DTT; 10% SDS; 250 mM Tris·HCL, pH 6.8; 30% glycerol; 0.02% bromophenol blue) followed by boiling at 95°C for 2 min. Proteins were separated by SDS-PAGE on a 10% gel, and PBPs were visualized using a Typhoon Trio (excitation: 488 nm, emission: 520 nm, GE Healthcare). As loading control, gels were stained with Coomassie Brilliant Blue G-250. The gel represents the results of two biologically independent determinations.

### Zymography

Cultures were grown as described for the muropeptide analysis. Autolysins were extracted from 10 mL culture with 4% SDS and protein concentrations were determined using the BCA protein assay (Pierce) with BSA as standard. 10 µg protein was separated by SDS-PAGE as previously described [27] in a resolving gel containing 0.1 mg/mL purified cell wall from USA300 grown in BHI. Following electrophoresis, gels were washed 3×15 min in water and 30 min in renaturation buffer (50 mM Tris-HCl (pH 7.5), 0.1% Triton X-100, 10 mM CaCl_2_, 10 mM MgCl_2_). The gels were then incubated in renaturation buffer at 37°C with gentle agitation for 20 hours, stained with 0.4% methylene blue, 0.01% KOH, 22% EtOH, and destained with water prior to photography. The gel represents the results of two biologically independent determinations.

### Measurement of autolysis

Unstimulated and Triton X-100 stimulated autolysis was measured as described by Gustafson *et al*. [28]. Briefly, cultures were grown to mid-exponential phase in the absence or presence of 16 µg/mL TDZ, harvested by centrifugation and washed twice with ice-cold Milli-Q water. Pellets were resuspended in 50 mM Tris-HCl (pH 7.5) buffer (unstimulated) or 50 mM Tris-HCl (pH 7.5) buffer containing 0.05% (wt/vol) Triton X-100 (stimulated) to an OD_580_ of 2.0, and incubated at 30°C with shaking. The decrease in OD_580_ was measured every 30 min. The data is presented as the mean of three independent experiments.

### Muropeptide analysis

An ON culture of USA300 was diluted to OD_600_ 0.005 in BHI without or with 16 µg/mL TDZ or 0.125 µg/mL DCX and grown to exponential phase (OD_600_ 0.8–1.0) at 37°C with aeration. To examine the effect of exogenous glycine, 10 mM extra glycine was added to the BHI from a sterile 2 M stock solution. Analysis of the UDP-linked precursor pool was performed as described by Gardete *et al*. [29]. Bacterial cell walls were prepared as described before [30] with the addition of a second SDS wash step to remove the RNase, DNase, and Trypsin. To remove the wall teichoic acids, 5 mg lyophilized cell wall was incubated with 1 mL 48% hydrofluoric acid for 48 hours at 4°C. PGN was recovered by centrifugation, washed three times with 100 mM Tris-HCl (pH 7), and then three times with water. Lyophilized PGN was digested with mutanolysin (Sigma), reduced, and analyzed by reverse-phase HPLC as described elsewhere [31]. Each chromatogram is representative of two biological replicates. Identification of the eluted peaks was done by comparison with the muropeptide HPLC profiles of reference strains previously analyzed [30]. Additionally, peaks of interest were collected, desalted using a Hypersil C18 ODS column, lyophilized, and analyzed by mass spectrometry. Samples were spotted directly onto the MALDI plate using 5 mg/mL α-ciano-4-hydroxycinnaminic acid in 50% (v/v) acetonitrile, 5% (v/v) formic acid as the matrix. Mass spectra were acquired in reflectron negative MS and MS/MS modes using a 4800 plus MALDI-TOF/TOF MS analyzer (PO25MS). Mass spectrometry data was provided by the Mass Spectrometry Laboratory, Analytical Services Unit, Instituto de Tecnologia Química e Biológica, Universidade Nova de Lisboa. Amino acid analysis of the purified PGN was performed as described in [32].

## Results

### TDZ sensitizes USA300 to inhibitors of late-stage PGN biosynthesis

**Figure 1:**
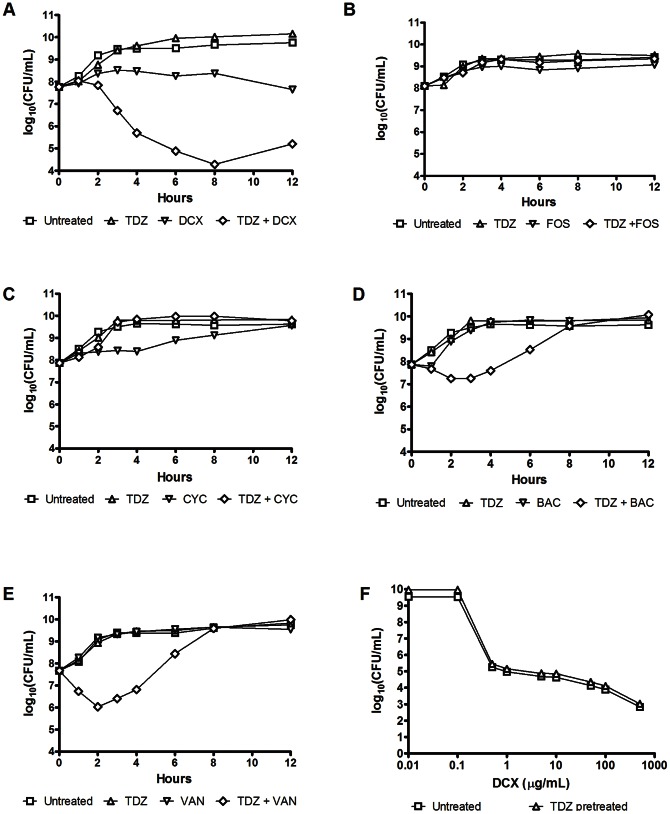
Impact of TDZ on the sensitivity to cell wall targeting antibiotics. (A-E) Time-kill assays showing the effect of TDZ (16 µg/mL, ¼×MIC) and (A) DCX (0.125 µg/mL, 1×MIC), (B) fosfomycin (FOS, 3 µg/mL), (C) D-cycloserine (CYC, 8 µg/mL), (D) bacitracin (BAC, 32 µg/mL) or (E) vancomycin (VAN, 1.5 µg/mL) on survival of USA300. (F) Susceptibility of post-exponential phase cultures grown in the absence or presence of TDZ (16 µg/mL) to DCX was determined by population analysis.

The ability of TDZ to potentiate the antimicrobial effect of dicloxacillin (DCX) against the epidemic CA-MRSA clone USA300 was examined in a time-kill assay. The strain was grown in liquid medium in the absence or presence of TDZ and/or DCX, and the viability of the bacteria was monitored by CFU counts (Figure 1A). The combination of TDZ (¼×MIC) and DCX (1×MIC) reduced the viability by 3 log_10_ CFU/mL compared to DCX alone after 8 hours of treatment, showing that TDZ sensitizes the cells to the action of DCX.

To clarify whether the effect of TDZ is specific for DCX, a series of time-kill assays were conducted examining the effect of TDZ combined with subinhibitory concentrations of other classes of cell wall targeting antibiotics. When TDZ was combined with fosfomycin or D-cycloserine, both of which target the cytosolic stage of PGN biosynthesis, cells were not further sensitized to the action of the antibiotics (Figure 1B-C). In fact, USA300 managed D-cycloserine treatment better in the presence of TDZ. Nor was a synergistic effect observed if TDZ was administered together with an inhibitory concentration of fosfomycin (6 µg/mL, data not shown). Interestingly, when TDZ was combined with bacitracin or vancomycin, which targets the later stages of PGN biosynthesis, an initial decrease in CFU counts was observed compared to either drug alone (Figure 1D-E). The decrease, however, was followed by an adaptation to the combined treatment after approximately 3 hours. Altogether, these results show that TDZ is able to sensitize the bacteria to several classes of antimicrobials with different targets in the late stages of PGN synthesis. Similarly, we observed that TDZ caused a 4-fold increase in the sensitivity of USA300 to the glycyl-glycine endopeptidase lysostaphin, reducing the MIC from 1 µg/mL in BHI to 0.25 µg/mL in BHI medium supplemented with 16 µg/mL TDZ (data not shown).

To investigate the prolonged effect of TDZ treatment on β-lactam sensitivity, a population analysis was conducted examining the DCX susceptibility after 8 hours of TDZ pretreatment. The results showed no difference between the profile of cells grown with or without TDZ (Figure 1F). Thus, TDZ did not select for a sensitive subpopulation or induce lasting changes in DCX susceptibility of the population and, consequently, both drugs must be administered simultaneously to obtain a synergistic effect.

### Changes in global gene expression induced by TDZ

In *S. aureus*, transcriptional profiling using DNA microarrays has previously been used to gain insight into the mode of action of antimicrobial agents [33]–[35]. We treated exponentially growing *S. aureus* cells with a subinhibitory concentration of TDZ (¼×MIC) alone or in combination with DCX (1×MIC) for 30 min and compared the transcriptional response to untreated cells using custom-made whole-genome oligoarrays (see Materials and Methods). Statistically significant 2-fold or greater changes were found for 188 up-regulated and 151 down-regulated genes following treatment with TDZ, whereas the combination of TDZ and DCX resulted in 204 up-regulated and 187 down-regulated genes (Table S2 and S3). Interestingly, no significant differences were found between TDZ and TDZ + DCX treatment, showing that the applied DCX concentration has no effect on the transcriptional response within the short time frame of the experiment, whereas TDZ induces a plethora of changes. The microarray data of 15 genes was validated by quantitative real-time PCR (RT-qPCR) analysis (Figure S2).

**Figure 2:**
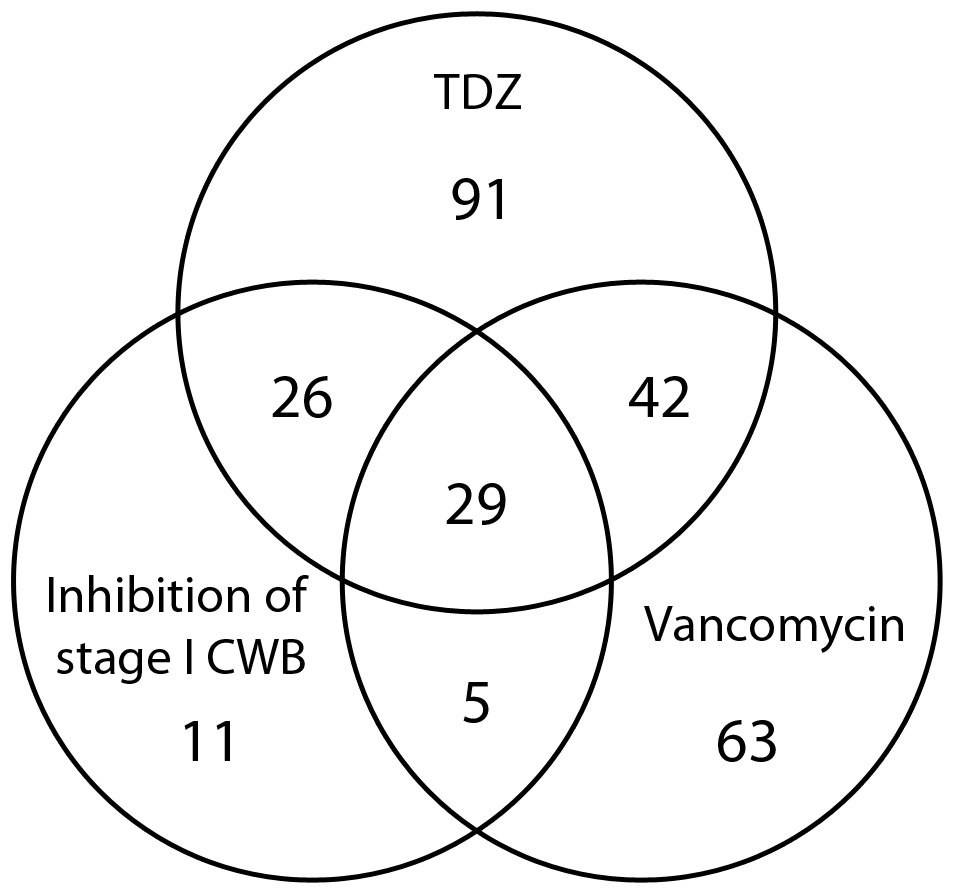
Comparison of the TDZ stimulon and genes induced by inhibition of PGN biosynthesis. Venn diagram showing the number of genes induced by TDZ, vancomycin [37], and inhibition of early cell wall synthesis (i.e. genes commonly induced in response to fosfomycin treatment, depletion of MurB, and depletion of MurE [36]).

We compared the transcriptional profile of TDZ treatment with the genome-wide transcriptional response of *S. aureus* to inhibition of PGN synthesis at different stages in the pathway (Table S2). Strikingly, 77% of the genes overexpressed in response to inhibition of early-stage PGN biosynthesis [36] were represented in the TDZ stimulon (Figure 2). Additionally, more than half of the genes that were previously found to be induced by vancomycin [37] were also induced in response to TDZ (Figure 2). On the contrary, a relatively small percentage of the D-cycloserine, bacitracin, and oxacillin stimulons (<30%) [38] overlapped the TDZ stimulon (Table S2). This pattern was also seen for genes that were down-regulated in response to the inhibitors, with 44% and 30% of the genes down-regulated in response to inhibition of early-stage PGN biosynthesis and vancomyin, respectively, found among the genes with decreased expression following TDZ treatment (Table S3). Collectively, these observations suggest that TDZ interferes with PGN synthesis, most likely at a stage that precedes the transpeptidation reaction catalyzed by the PBPs.

The group of genes commonly induced by TDZ, by inhibition of the cytosolic stage of PGN biosynthesis, and/or by vancomycin included genes involved in synthesis of glutamate (*gltBD*), lysine (*lysC-asd-dapABD, dhoM*), branched-chain amino acids (BCAAs) (*ilvDBNC-leuABCD-ilvA*), and histidine (*hisGDCBHAFEI*) as well as amino acid/oligopeptide transporters (*oppBCDFA*, *SA2396*). In rich medium, the amino acid requirements of *S. aureus* are met by uptake from the environment rather than *de novo* synthesis; consequently, genes involved in amino acid biosynthesis are repressed [39], [40]. The induction of these genes following inhibition of PGN biosynthesis may be regarded as an attempt to supply the essential precursors for the pathway. CodY is a well-characterized repressor of amino acid biosynthesis and transport genes responding to the intracellular levels of BCAAs and GTP in *S. aureus* and related Gram-positive species [41]–[43]. A comparison between the transcriptional response to TDZ and the transcriptional profile of a Δ*codY* mutant revealed that 40% of the genes belonging to the CodY regulon were in fact induced in response to TDZ (Table S2).

The two-component system VraSR is known to respond to inhibition of PGN biosynthesis and cell wall damage by inducing a 46 membered regulon including its own operon [37], [44]. Following TDZ treatment, we found that 27 VraSR-controlled genes were induced and among the most highly up-regulated genes in the transcriptome (Table S2). This observation further supports the hypothesis that TDZ either directly or indirectly compromises the cell wall integrity. VraSR-regulated genes induced by TDZ included amongst others genes associated with PGN synthesis and response to cell wall targeting antibiotics (*murZ, fmtA, sgtB,cwrA, tcaA, drp35*) as well as genes involved in protein degradation and modification (*spsA, ctpA,htrA_1_, prsA*).

Among the genes down-regulated by TDZ, functional categories significantly overrepresented as determined by Fisher's exact test comprised cell envelope proteins and transport/binding proteins (Table S3) many of which remain uncharacterized. Interestingly, most of the surface and extracellular proteins previously found to be induced by the SaeRS two-component system [45] were among the genes most down-regulated in response to TDZ (*coa, SA0743, SA1000, efb, SA1004, sbi, fnbB, set15, SA0394, nuc, hla, hlgC*). Additional genes known or expected to contribute to virulence (*ebhA, sasG, fnbA, aur, hlgAB, sarS*) including several iron transporters (*sirABC, sbnBC, sstA*) were also found to be down-regulated in response to TDZ.

### TDZ affects the ultrastructure of the cell wall

**Figure 3:**
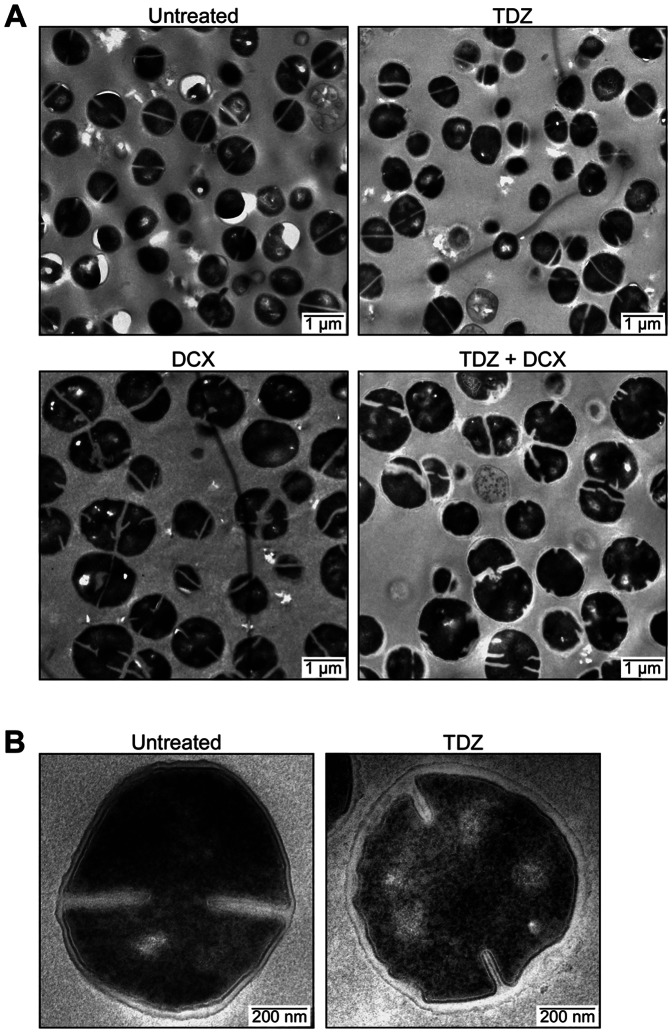
Morphology of USA300 following treatment with TDZ and/or DCX. (A and B) Cultures were subjected to the drugs for 2.5 hours as described in Materials and Methods and imaged by transmission electron microscopy of thin sections.

To examine if TDZ treatment leads to morphological changes of the cells, USA300 was treated with TDZ and/or DCX for 2.5 hours in the exponential growth phase and imaged by TEM. Results showed that DCX treatment alone or in combination with TDZ gave rise to enlarged cells with severe defects in cell division and septum formation whereas TDZ treated cells resembled the untreated control regarding cell size and septation (Figure 3A). However, a closer inspection of the TDZ treated cells revealed a thickened, irregular cell wall giving rise to a wavy appearance of the cell envelope (Figure 3B).

Fluorescence microscopy using the cell wall stain Van-FL and DNA stain Hoechst 33342 confirmed the interference of DCX with cell division and showed that chromosome segregation and cell division was unaffected by TDZ even after prolonged treatment (Figure S3A). Van-FL binds the D-alanyl-D-alanine carboxy termini of PGN, staining the entire cell wall and septum [46], [47] as evident for the untreated culture. Here, the peripheral cell wall was uniformly stained, whereas the signal was more intense in the septal wall where synthesis of new PGN takes place. Following growth in the presence of TDZ, staining of the peripheral cell wall was less consistent and intensely stained spots or hemispheres were observed.

Concentrations of phenothiazines around 40–50 µM, similar to the concentration of TDZ applied here, have been demonstrated to permeabilize the cytoplasmic membrane of human platelets to small solutes such as adenine nucleotides [48], [49]. To examine if a similar phenomenon takes place when *S. aureus* is treated with TDZ or DCX, cells were stained with the DNA stain propidium iodide (PI) and visualized by fluorescence microscopy. The inability of PI to penetrate untreated or TDZ treated cells confirmed the membrane integrity of these cells, whereas DCX treatment gave rise to a large population of cells with compromised cell envelopes (Figure S3B).

### Effect of TDZ on PBP and autolysin profiles

**Figure 4:**
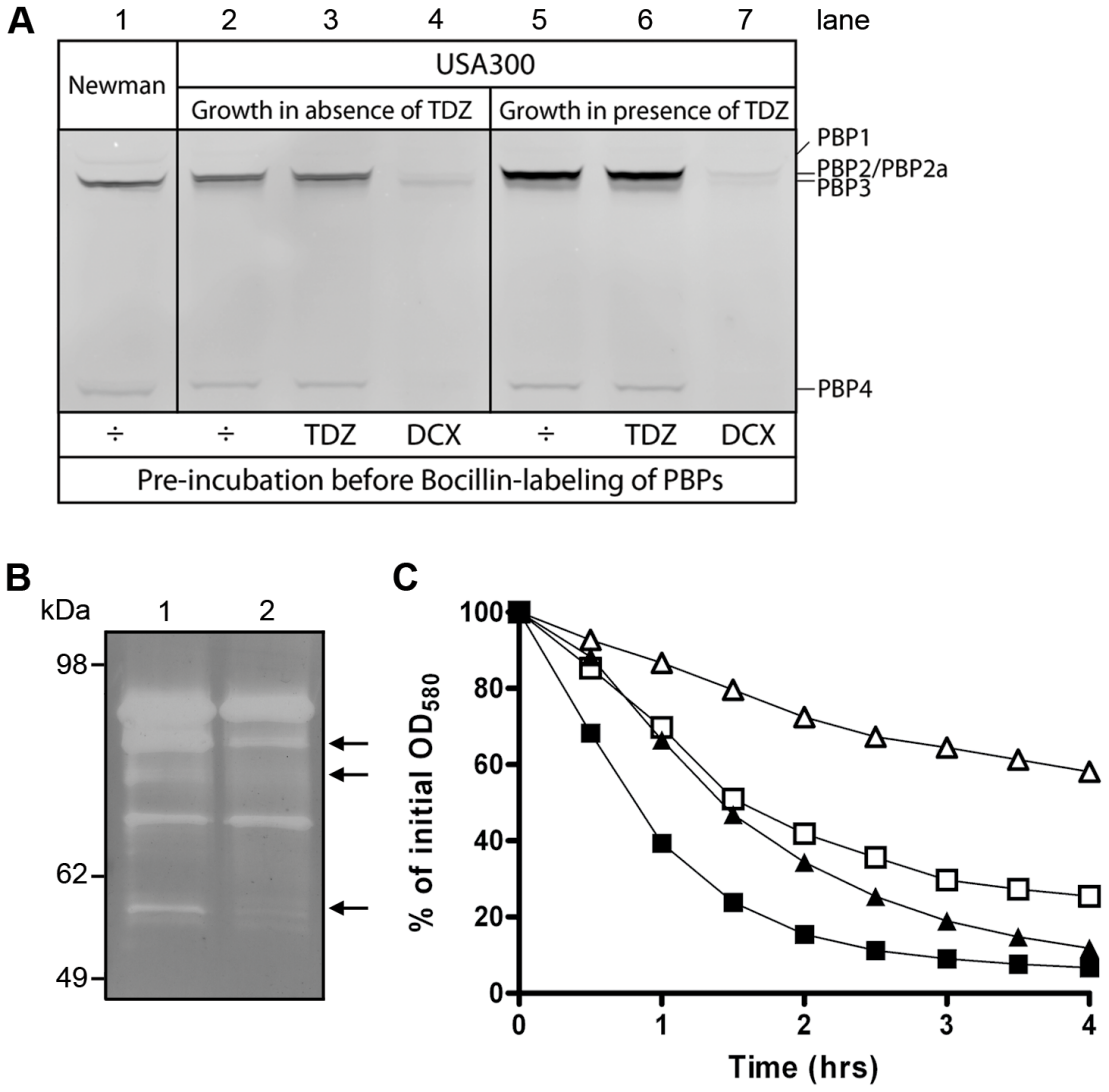
Effect of TDZ on PBPs and autolysis. (A) Membrane fractions were isolated from USA300 grown to mid-exponential phase in the absence or presence of TDZ (16 µg/mL) and pre-incubated with 16 µg/mL TDZ, 50 µg/mL DCX, or assay buffer. PBPs were labeled with Bocillin-FL, separated by SDS-PAGE, and visualized by fluorography. The PBP profile of MSSA strain Newman is shown for comparison. (B) Zymogram analysis of autolysins extracted from the cell walls of USA300 grown to mid-exponential phase in the absence (1) or presence (2) of 16 µg/mL TDZ. 10 µg or protein extract was separated in a 10% SDS-polyacrylamide gel containing purified cell walls from USA300 grown in BHI. Arrows indicate bands with decreased intensity following TDZ treatment. (C) Unstimulated and Triton X-100 stimulated autolysis of USA300 grown to mid-exponential phase in the absence (squares) and presence (triangles) of 16 µg/mL TDZ. Unstimulated autolysis is represented by open symbols, and Triton X-100 stimulated autolysis by closed symbols.

To assess whether TDZ affects the function or levels of the PBPs, and consequently the synthesis of the bacterial cell envelope, Bocillin-FL, a fluorescence-conjugated penicillin V derivative, was used to label the PBPs in membranes prepared from USA300 grown in the absence or presence of 16 µg/mL TDZ (Figure 4A). Preincubation of membranes with DCX, which blocks the active site of sensitive PBPs and reduces the Bocillin-FL signal (lane 4 and 7) was included as a control, and so was the PBP profile of MSSA strain Newman (lane 1). Preincubation of membranes with TDZ prior to Bocillin-FL labeling did not affect the intensity of the signal from the PBPs (Figure 4A lane 2 vs. 3, lane 5 vs. 6, and Figure S4) showing that TDZ neither blocks the transpeptidase domains nor promotes the binding of β-lactam antibiotics to the PBPs. Following growth in the presence of TDZ, a stronger signal from the band containing PBP2, PBP2a, and PBP3 was observed (lane 2 vs. 5), whereas there was no significant effect on PBP1 and PBP4. As TDZ does not affect the binding of Bocillin-FL, the changes in signal intensity must reflect changes in the protein quantities.

Cells with compromised cell walls, that appear when *S. aureus* is grown in the presence of TDZ, could result from an altered level or activity of autolysins, enzymes capable of degrading the bacterial PGN, leading to lysis of bacteria. Previously, the activity of *S. aureus* autolysins was shown to be regulated in response to perturbations of cell wall synthesis and to contribute to methicillin resistance [50], [51]. To examine if TDZ treatment affects the autolysins, cell wall proteins from USA300 grown in the absence and presence of TDZ were extracted and analyzed by zymography (Figure 4B). Several bands of lower intensity were observed following TDZ treatment, indicating a decrease in quantity or activity of murein hydrolases in the cell wall. The autolytic properties were furthermore assayed by measuring the autolysis rate of unstimulated and Triton X-100 stimulated static cultures (Figure 4C), which confirmed that growth in the presence of TDZ leads to decreased autolytic activity of USA300.

### TDZ-induced changes in the muropeptide composition of the PGN

Intracellular PGN precursors are known to accumulate in *S. aureus* in response to vancomycin treatment or inhibition of early cell wall synthesis [29], [52]–[54]. The resemblance between the transcriptional response to TDZ treatment and these conditions prompted us to examine the cytoplasmic pool of UDP-linked cell wall precursors; however, no accumulation was observed when cells were treated with 16 µg/mL TDZ (data not shown).

**Figure 5:**
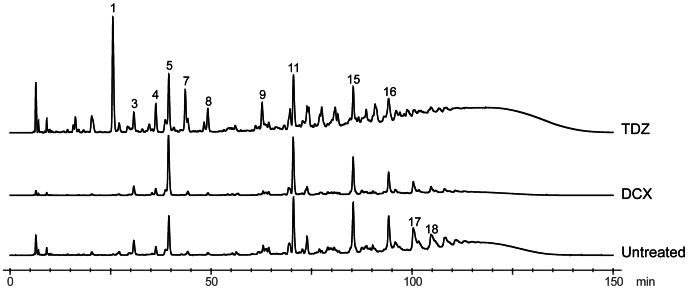
Effect of TDZ and DCX on the muropeptide composition of USA300 PGN. PGN was isolated from cultures grown to exponential phase in the absence or presence of TDZ or DCX and muropeptide compositions were analyzed by HPLC as described in Materials and Methods. Peak numbers are assigned according to [30]. The identity of peak 1, 4, 5, 7, and 11 was confirmed by mass spectrometry. Chromatograms are normalized to peak 11.

To characterize the effect of TDZ on the cell wall in more detail, we isolated PGN from USA300 grown in the presence or absence of TDZ or DCX and analyzed its muropeptide composition by HPLC (Figure 5). The HPLC profile of USA300 grown in BHI revealed the typical pattern of *S. aureus* with the highest peak found in the dimeric fraction (peak 11). Strikingly, TDZ treatment resulted in the appearance of several peaks that were either absent or found only in minor amounts in PGN from the untreated culture. Major peaks were purified, desalted, and analyzed by mass spectrometry to confirm their identity (Figure S5). Most notably, there was a large accumulation of the monomeric disaccharide pentapeptide without the pentaglycine branch (peak 1), and a smaller increase in monomers carrying an incomplete cross-bridge with a single glycine (peak 4). Furthermore, monomers carrying an alanine in the interpeptide cross-bridge were also more frequent (peak 7). The additional peaks with retention times above 60 min most likely represent multimeric combinations of the normal pentaglycine substituted precursor and the various alternative precursors. The decrease in glycine and increase in alanine content of the PGN was confirmed by amino acid analysis (data not shown). The fact that nonsubstituted pentapeptides, which cannot be used efficiently for cross-linking [55], accumulate following TDZ exposure, may explain the reduction in the oligomeric fraction observed following TDZ treatment (Table 1).

**Table 1:** Percent muropeptides and overall degree of cross-linking in untreated, TDZ, and DCX treated MRSA USA300.

Culture	Monomer*	Dimer*	Trimer*	Oligomer*	CL (%)**
Untreated	7.5	13.5	14.0	65.1	74.7
TDZ	14.6	11.8	12.6	61.0	69.2
DCX	17.7	19.1	16.0	47.2	62.8

* Peak areas of the indicated fractions were added and calculated in percent.

** The cross-linking (CL) value was calculated as follows: 0.5× dimer (%)+0.67× trimer (%)+0.9×oligomer (%) [90].

### TDZ-induced changes in S. aureus are complemented by the presence of increasing concentration of glycine in the growth medium

**Figure 6:**
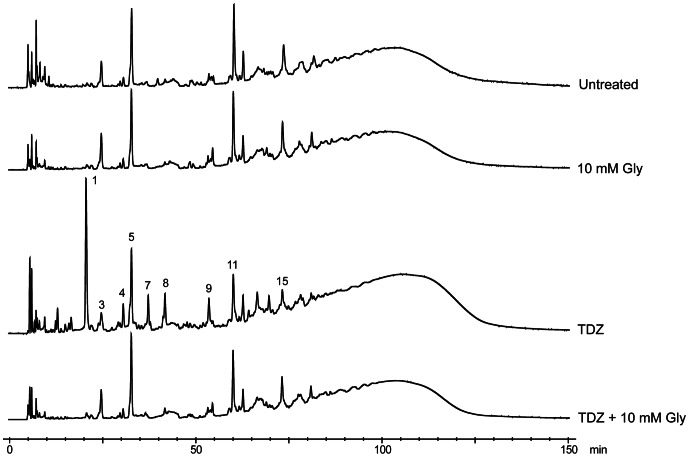
Exogenous glycine relieves the TDZ-induced effects on the muropeptide composition of USA300. *S. aureus* cells were grown to exponential phase in BHI medium or BHI medium supplemented with 10 mM glycine, in the absence or presence of 16 µg/mL TDZ. PGN was isolated from the cultures and muropeptide compositions analyzed by HPLC as described in Materials and Methods. Peaks were identified as described for Figure 5. Chromatograms are normalized to peak 11.

As phenothiazines in general have been shown to interfere with various membrane-based processes such as glycine uptake [17], [18], we hypothesized that the accumulation of unbranched muropeptides could be a consequence of the inability to import glycine from the growth medium in the presence of TDZ. If that was the case, the deleterious effect of TDZ might be compensated by the addition of glycine to the growth medium. To examine this hypothesis, the effect of TDZ on the muropeptide composition of USA300 grown in BHI supplemented with glycine was analyzed by HPLC. High concentrations of exogenous glycine have previously been demonstrated to give rise to stem peptides with D-Ala-Gly termini and reduce the methicillin-resistance of MRSA [56], thus, to avoid this modification we supplemented the medium with 10 mM glycine, which did not significantly change the muropeptide profile in comparison to BHI alone (Figure 6). Remarkably, addition of 10 mM glycine together with TDZ completely abrogated the incorporation of modified muropeptides in the PGN that was caused by TDZ alone, giving rise to a profile that was indistinguishable from profiles derived from BHI and BHI +10 mM glycine (Figure 6).

To investigate the significance of exogenous glycine in relation to the effect of TDZ on the DCX sensitivity of USA300, the viability assay presented in Figure 1A was repeated in BHI supplemented with 10 mM glycine. If the changes in the muropeptides caused by TDZ are directly responsible for the increased DCX sensitivity of USA300 in the presence of TDZ, we would expect to see a protective effect of exogenous glycine against the TDZ-mediated killing by DCX of *S. aureus*. As shown in Table 2, the addition of glycine to the growth medium, together with TDZ and DCX, increased the viability with 1 log_10_ CFU/mL compared to TDZ + DCX alone. Thus, exogenous glycine indeed offered a modest protection against the effect of TDZ on DCX sensitivity. Collectively, these results suggest that the reduction in DCX resistance caused by TDZ may be explained, at least in part, by alterations in the muropeptide composition of *S. aureus*.

**Table 2:** The effect of exogenous glycine on the TDZ-induced sensitivity to DCX in USA300.

Exogenous glycine	Untreated	TDZ	DCX	TDZ + DCX
−	2.7E+9	4.3E+9	2.0E+8	1.6E+5
+	4.4E+9	5.0E+9	2.5E+8	1.6E+6

Time-kill assays were performed by adding TDZ (16 µg/mL, ¼×MIC) and/or DCX (0.125 µg/mL, 1×MIC) to USA300 grown in BHI medium as described in Materials and Methods. Glycine (10 mM) was added to the cultures simultaneously with the addition of the antimicrobial drugs. The results presented correspond to CFU/mL measured at 8 hours after the addition of glycine, TDZ and/or DCX.

## Discussion

Methicillin-resistant *Staphylococcus aureus* have acquired an additional PBP (PBP2a) with low affinity to β-lactams; the resistance level, however, is influenced by native factors, many of which are involved in cell wall synthesis and architecture [57]–[59]. Agents that suppress β-lactam resistance by perturbing the functions of such auxiliary factors are gaining interest as potential adjuvants for combined treatment with β-lactam antibiotics against MRSA infections. Many natural products including manuka honey and green tea are known to potentiate the effect of oxacillin against MRSA [60], [61]; in the latter case, the synergistic activity has been ascribed to polyphenolic compounds of which epicatechin gallate (ECg) is the most potent [62]. ECg was shown to bind the cell membrane of MRSA causing a change in fluidity and fatty acid composition and in turn delocalization of PBP2 from the septum [35]. Several reports have established the functional cooperation between enzymes involved in PGN synthesis in *S. aureus* and the requirement of PBP2 transglycosylase (TGase) activity to express β-lactam resistance [63]–[65]; thus, displacement of PBP2 from the division site is expected to greatly reduce the oxacillin tolerance of MRSA. A comparable mode of action has been proposed for the polyamine spermine; recent evidence supports a direct interaction between spermine and PBP2 that presumably weakens the interaction between PBP2 and other enzymes involved in PGN synthesis and/or inhibits its TGase activity, thus rendering MRSA susceptible to oxacillin [66]. The importance of the cooperative action between PBPs for expression of β-lactam resistance is further underscored by studies demonstrating synergy between inhibitors of early wall teichoic acid (WTA) synthesis and β-lactam antibiotics [67], [68]. In the absence of WTAs, the high-level cross-linking that characterizes *S. aureus* PGN is eliminated due to delocalization of PBP4 from the septum [69]. This effect in combination with β-lactams that target the transpeptidation activity of PBP2 has proved to be lethal to MRSA [68]. Recently it was shown that β-lactam resistance of MRSA depends on β-*O*-GlcNAcylation of WTA [70], suggesting that this particular modification is involved in PBP4 localization. Thus, understanding the mechanisms that underlie methicillin resistance can aid the identification and optimization of additional helper compounds that once again enable the clinical use of β-lactams against MRSA. In the present study we explored the effects of TDZ, a promising candidate for combined treatment with β-lactam antibiotics, on USA300. TDZ itself has antimicrobial activity [71], but here we show that a subinhibitory concentration of TDZ has major impact on the cell wall, providing new insights into the mechanism underlying the TDZ-mediated resensitization of MRSA to DCX.

Imaging of cells grown in the absence or presence of TDZ by electron microscopy revealed that the latter generally had thicker cell walls and the cell wall thickness of the individual cells was very irregular. Cell wall thickening is a hallmark of VISA (vancomycin intermediate *S. aureus*) strains and arise due to decreased autolytic activity and in some cases increased PGN synthesis [72]. We observed a lower autolytic activity following growth in the presence of TDZ, however, to our knowledge, VISA strains displaying irregular cell wall thickness similar to the TDZ treated cells have not been described. In a series of time-kill assays, we furthermore demonstrated that TDZ increases the sensitivity of USA300 to several antimicrobial agents targeting the later stages of PGN biosynthesis, including vancomycin. Increased vancomycin sensitivity in the presence of TDZ has previously been reported for vancomycin-resistant *Enterococcus faecalis* and *E. faecium* isolates [73], [74], thus altogether the data does not seem to support the induction of a VISA phenotype following TDZ treatment of USA300.

Using a transcriptomic approach, we have demonstrated that TDZ causes major changes in gene expression, many of which can be related to interference with PGN biosynthesis. Most prominently, a large overlap between the TDZ stimulon and genes induced by inhibition of early cell wall biosynthesis (i.e. genes commonly induced by inhibition/depletion of MurA/MurZ, MurB, and MurE [36]) or vancomycin treatment [37] was observed, which suggests that TDZ could be affecting a step in the PGN pathway which lies before the transpeptidation reaction performed by the PBPs. Previously, inhibitors of PGN synthesis targeting stages prior to the β-lactams were shown to reduce the methicillin-resistance of MRSA [75] similar to the effect of inactivating the enzymes catalyzing these steps [52], [53], [55], [76], [77]. Thus, interference of TDZ with an earlier step in PGN biosynthesis could explain the increased sensitivity of MRSA to DCX. The global transcriptional response to D-cycloserine, another inhibitor of the intracellular stage of the PGN pathway, has previously been determined [38], but the resemblance between this stimulon and the TDZ stimulon was somewhat smaller than for the conditions mentioned above. However, as D-cycloserine prevents the formation of D-alanine, it interferes with synthesis of both PGN and teichoic acids, which may explain the different transcriptomic responses.

The transcriptomic analysis furthermore revealed that upon addition of TDZ, part of the CodY regulon was derepressed and genes involved in amino acid biosynthesis/uptake as well as the tricarboxylic acid (TCA) cycle, which supplies precursors for amino acid biosynthesis, were induced. This could indicate that TDZ exposure leads to an intracellular amino acid shortage. In *S. aureus* the phenothiazine prochlorperazine was shown to reduce the transmembrane potential, but not ΔpH [8], and since many bacteria utilize secondary transporters for amino acid uptake [78] it is likely that a similar effect of TDZ would have consequences for the rate of amino acid transport, thus necessitating *de novo* synthesis. In support of this notion, the expression of several sodium-dependent transporters was reduced by TDZ, whereas mainly ABC-type transporters were induced. CodY has previously been described as a link between metabolism and virulence in several Gram-positive species [41], [42], [79]. However, despite the derepression of CodY-controlled genes involved in metabolism in response to TDZ, most of the virulence related genes controlled by CodY were unaffected or even downregulated (*hla, SA1000, sbi, cap5FG, efb, lrgB, nuc, SA1725*) by TDZ. This observation suggests that derepression of the virulence genes is not sufficient to stimulate their expression and that positive regulators (e.g. the SaeRS two-component system [45]) may be necessary to induce transcription.

In line with the transcriptomic analysis, we found that growth in the presence of TDZ leads to striking differences in the muropeptide composition compared to the normal profile of *S. aureus*. Interference with formation of the normal pentaglycine interpeptide was evident from the incorporation of unsubstituted monomers and monomers carrying an alanine or a single glycine in the interpeptide. Formation of the interpeptide takes place at the inner surface of the membrane by sequential addition of glycine by FemX (Gly_1_), FemA (Gly_2–3_), and FemB (Gly_4–5_) to the lipid-linked PGN precursor Lipid II [55], [77], [80]. Each of the cytoplasmic Fem enzymes have been proposed to use a dedicated non-proteinogenic tRNA^Gly^ as Gly donor, which is charged with glycine by the glycyl tRNA synthetase (GlyRS) encoded by *glyS* [80], [81]. Interestingly, our muropeptide analysis of USA300 treated with TDZ in the presence of 10 mM exogeneous glycine gave rise to a profile which was indistinguishable from the normal profile of *S. aureus*. Thus, it seems unlikely that the alterations in the PGN caused by TDZ alone are due to interference with the FemXAB enzymes. The *glyS* gene, which is regulated by a T-box system that responds to uncharged tRNA^Gly^ [82], [83], was induced by TDZ in our microarray analysis. Thus, we hypothesize that the changes in the muropeptide composition caused by TDZ results from a reduction of the intracellular level of glycine, which would lead to an increase in uncharged tRNA^Gly^, although a direct interference with the function of GlyRS cannot be ruled out at this point. Interestingly, the presence of exogenous glycine also resulted in a modest decrease in the TDZ-induced sensitivity to DCX. Taken together we propose that TDZ exposure leads to a decrease in the intracellular level of glycine which inhibits the production of normal PGN precursors with pentaglycine branches. The resulting decrease in normal building blocks for PGN synthesis may enhance the sensitivity of *S. aureus* to the β-lactam antibiotic DCX as well as other late-stage inhibitors of cell wall synthesis. Curiously, a similar effect was recently suggested for the non-antibiotic Cyslabdan, which enhances the potency of β-lactams against MRSA by inhibiting pentaglycine interpeptide bridge synthesis, most likely by direct binding to FemA [84]. Thus, the biosynthetic pathway for the pentaglycine interpeptide bridge appears to be a promising target for potentiating the effect of β-lactam antibiotics against *S. aureus*.

Interference with a wide range of membrane-based processes by TDZ and other phenothiazines has been well established in a range of human cell types and bacterial species [12]–[18]. The phenothiazines are known to interact preferentially with negatively charged phospholipids and partition into lipid bilayers near the polar/hydrophobic interface [10], [85]–[87], which is likely to impact the function of membrane proteins and membrane-associated factors. In line with this, we speculate that TDZ mainly exerts its effect on USA300 by embedding in the cytoplasmic membrane of *S. aureus*, though a direct interaction remains to be proven. As argued above, a major consequence of this putative membrane interaction could be reduced amino acid uptake from the environment, which may affect the PGN biosynthesis pathway indirectly by limiting the availability of substrates for precursor assembly. In future studies, it will be interesting to determine how the lipid bilayer of *S. aureus* is affected by TDZ and its consequences for membrane based processes (e.g. amino acid transport). Additionally, it will be important to clarify the connection between amino acid uptake/biosynthesis and β-lactam resistance in *S. aureus*, both *in vitro* and in an infection model.

In the present report, we have demonstrated that a subinhibitory concentration of TDZ has widespread effects on the cell wall, transcriptome, and antibiotic resistance of MRSA USA300. Although the concentration of TDZ used in this study is beyond the clinically achievable concentration in human plasma (0.5–1.0 µg/mL) [88], research in the mode of action of TDZ contributes to understanding the complex mechanisms underlying expression of β-lactam resistance in MRSA. In the future, this may contribute to the identification of novel antimicrobial targets or helper compounds with increased potency that, in combination with traditional antibiotics, can meet the challenge posed by the endemic MRSA strains. Moreover, the observation that expression of virulence genes like coagulase and fibrinogen-binding proteins, which disguise the bacterium from the host immune system, was decreased following TDZ treatment suggests that TDZ may assist phagocytosis of invading *S. aureus* bacteria. As TDZ is concentrated in macrophages and promotes the killing of intracellular bacteria [89], the drug may have dual functions, which altogether further the clearance of MRSA infections.

## Supporting Information

Figure S1 **Chemical structure of TDZ.** The chiral center of the molecule is marked by an asterisk.
(TIF)

Figure S2 **Verification of microarray data by RT-qPCR.**
(TIF)

Figure S3 **Fluorescence microscopy.** USA300 was grown in BHI broth for 7–8 generations to mid-exponential phase in the absence and presence of 16 µg/mL TDZ or 0.125 µg/mL DCX. (A) Cells stained with the cell wall dye Van-FL (1 mg/L) and Hoechst 33342 (1 mg/L). Scale bars correspond to 1 µm. (B) Membrane integrity examined by staining cells with the LIVE/DEAD BacLight Bacterial Viability Kit.
(TIF)

Figure S4 **Bocillin-FL labeling of PBPs.** Membranes isolated from USA300 grown in BHI broth were labeled with Bocillin-FL as described in Materials and Methods. Prior to Bocillin-FL labelling, membranes were preincubated with assay buffer (without TDZ) or increasing concentrations of TDZ.
(TIF)

Figure S5 **Analysis of the muropeptide composition of the cell wall PGN of** ***S. aureus*** **USA300 strain grown in the presence of TDZ.** (A) HPLC profiles of muropeptides released by mutanolysin digestion of the PGN purified from USA300, grown in the presence of TDZ (TDZ) or in its absence (BHI). Peaks were labeled from 1 to 18 according to [30]. In the presence of TDZ, there was accumulation of monomeric muropeptides, lacking the pentaglycine bridge (peak 1) or with an abnormal monoalanine bridge (peak 7). (B) Mass spectrometry analysis of different muropeptide peaks allowed their identification (shown as molecular structures). The expected and the observed masses are also shown. For clarity of the representation, each aminoacid is colored differently (alanine – red, glutamine – green, lysine – orange, glycine – black).
(TIF)

Table S1 **Primers used in this study.**
(PDF)

Table S2 **Genes upregulated by TDZ exposure.**
(PDF)

Table S3 **Genes downregulated by TDZ exposure.**
(PDF)
