# Supplementary figures and images for: Thioridazine Induces Major Changes in Global Gene Expression and Cell Wall Composition in Methicillin-Resistant Staphylococcus aureus USA300
Source: PLoS One. 2013 May 17;8(5):e64518. doi: 10.1371/journal.pone.0064518 (PMC3656896; doi:10.1371/journal.pone.0064518)

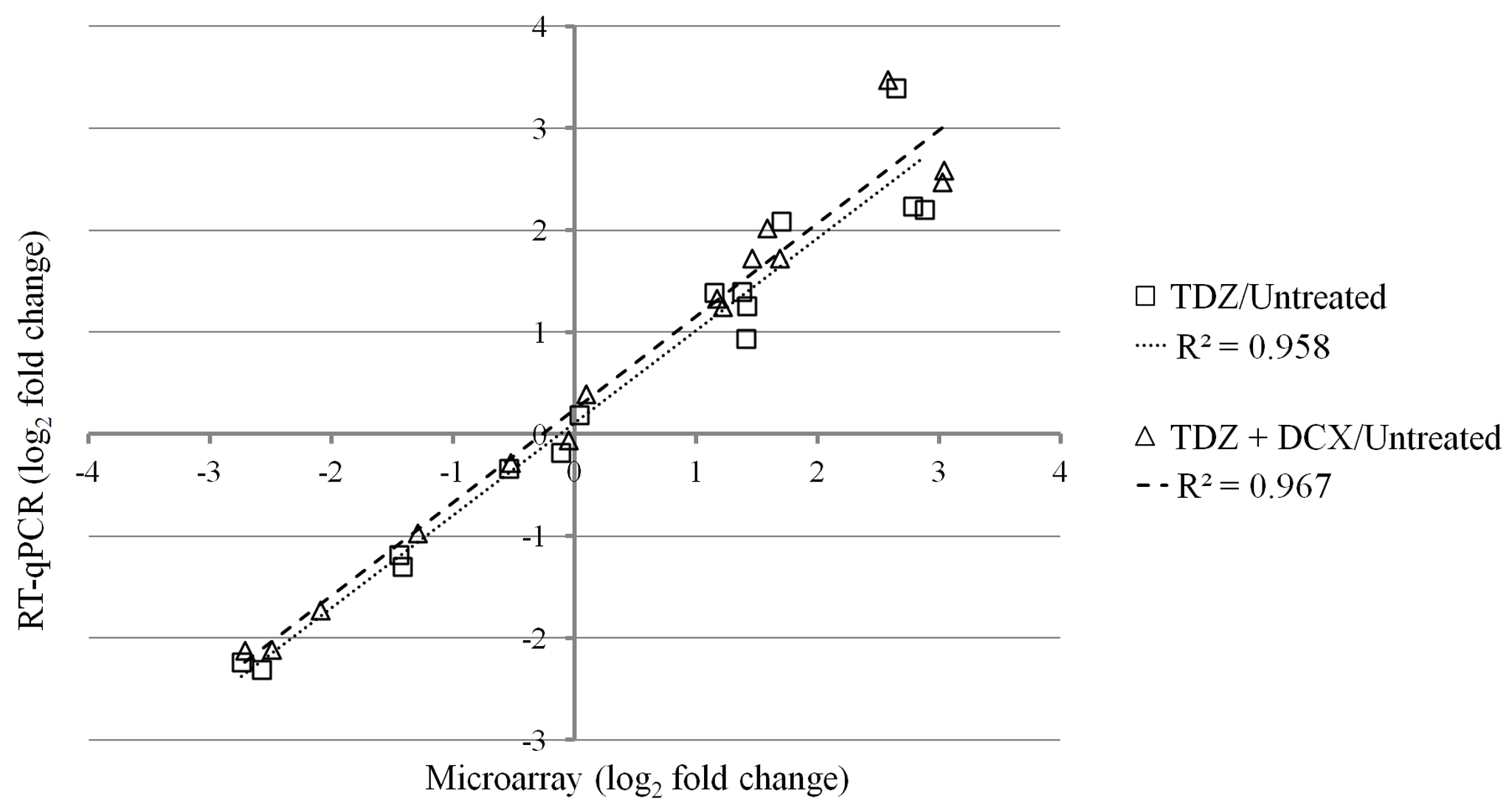

Supplement: Figure S2 — Verification of microarray data by RT-qPCR. (TIF) [file pone.0064518.s002.tif]

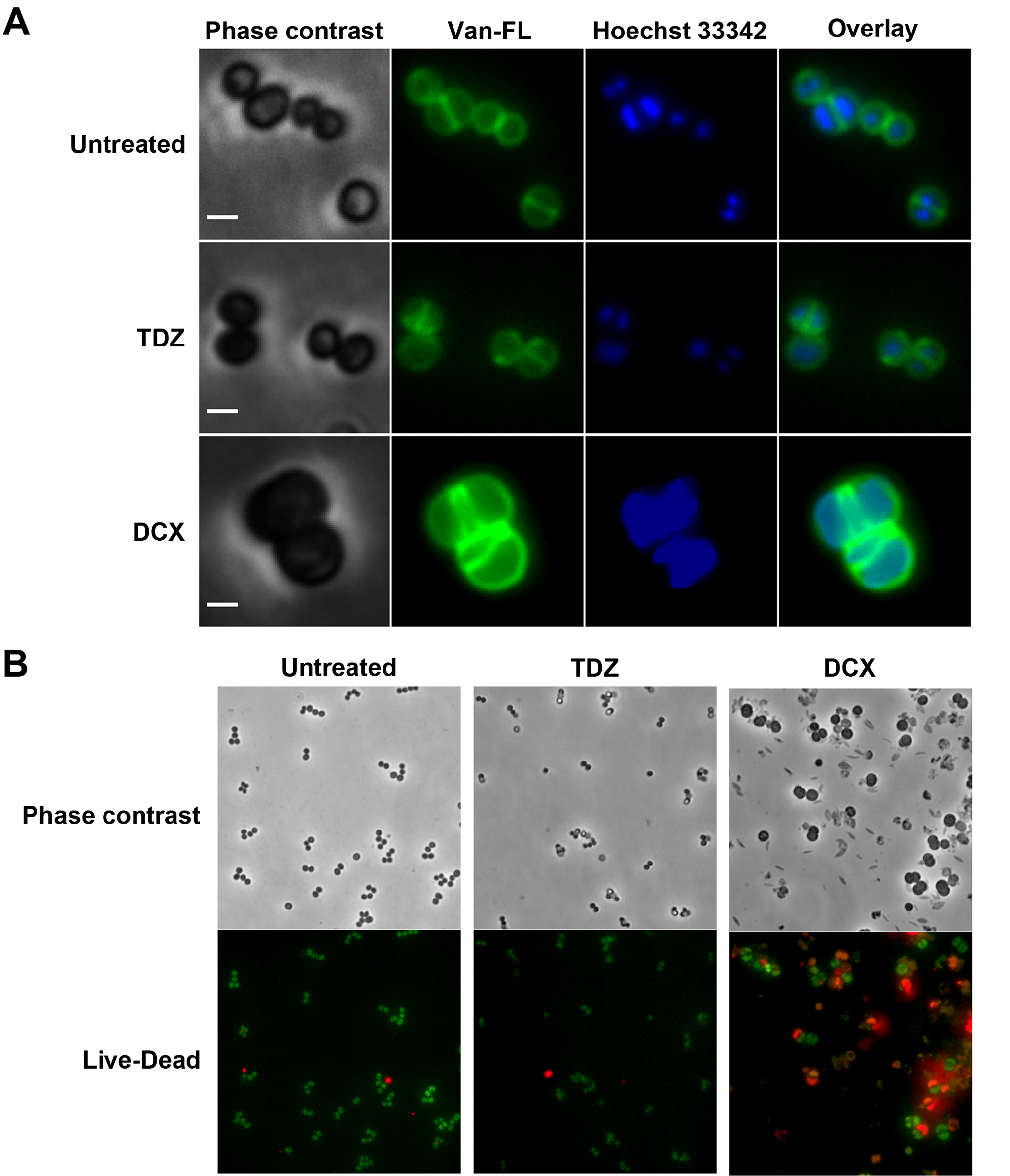

Supplement: Figure S3 — Fluorescence microscopy. USA300 was grown in BHI broth for 7–8 generations to mid-exponential phase in the absence and presence of 16 µg/mL TDZ or 0.125 µg/mL DCX. (A) Cells stained with the cell wall dye Van-FL (1 mg/L) and Hoechst 33342 (1 mg/L). Scale bars correspond to 1 µm. (B) Membrane integrity examined by staining cells with the LIVE/DEAD BacLight Bacterial Viability Kit. (TIF) [file pone.0064518.s003.tif]

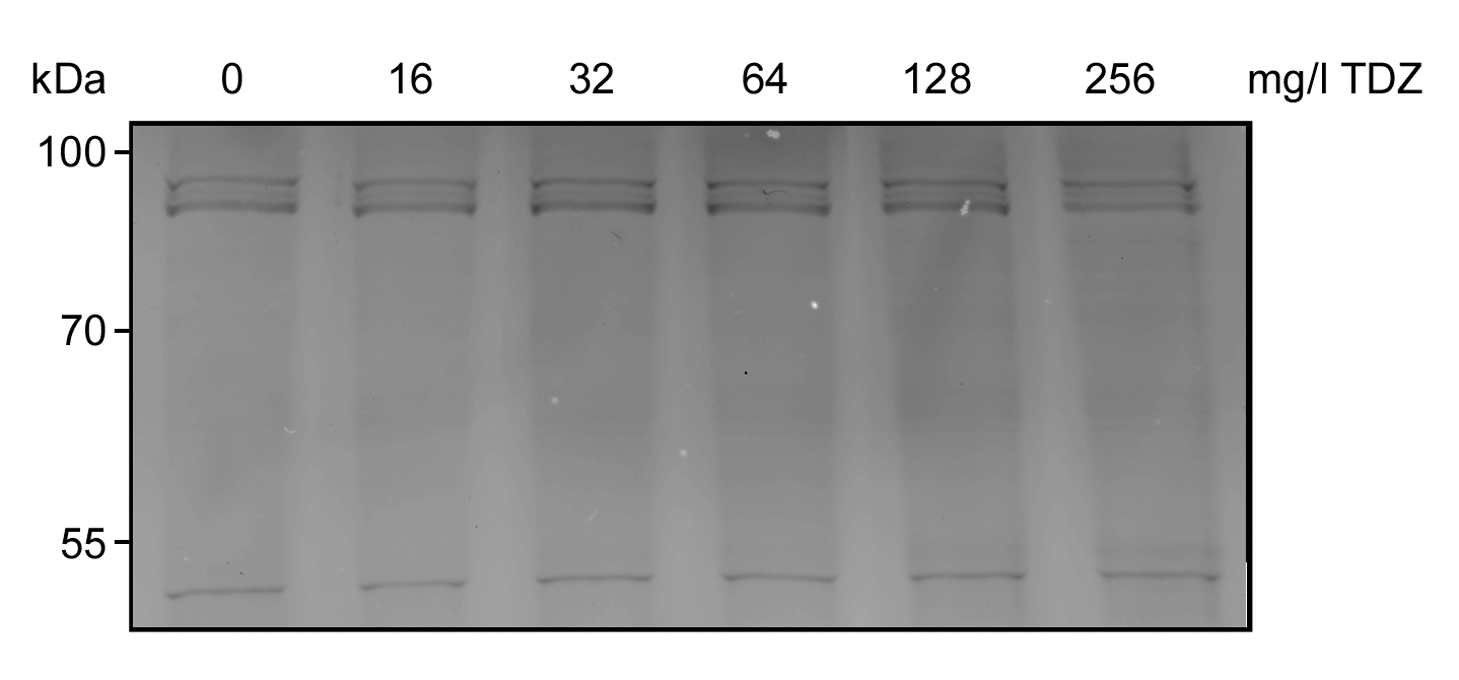

Supplement: Figure S4 — Bocillin-FL labeling of PBPs. Membranes isolated from USA300 grown in BHI broth were labeled with Bocillin-FL as described in Materials and Methods. Prior to Bocillin-FL labelling, membranes were preincubated with assay buffer (without TDZ) or increasing concentrations of TDZ. (TIF) [file pone.0064518.s004.tif]

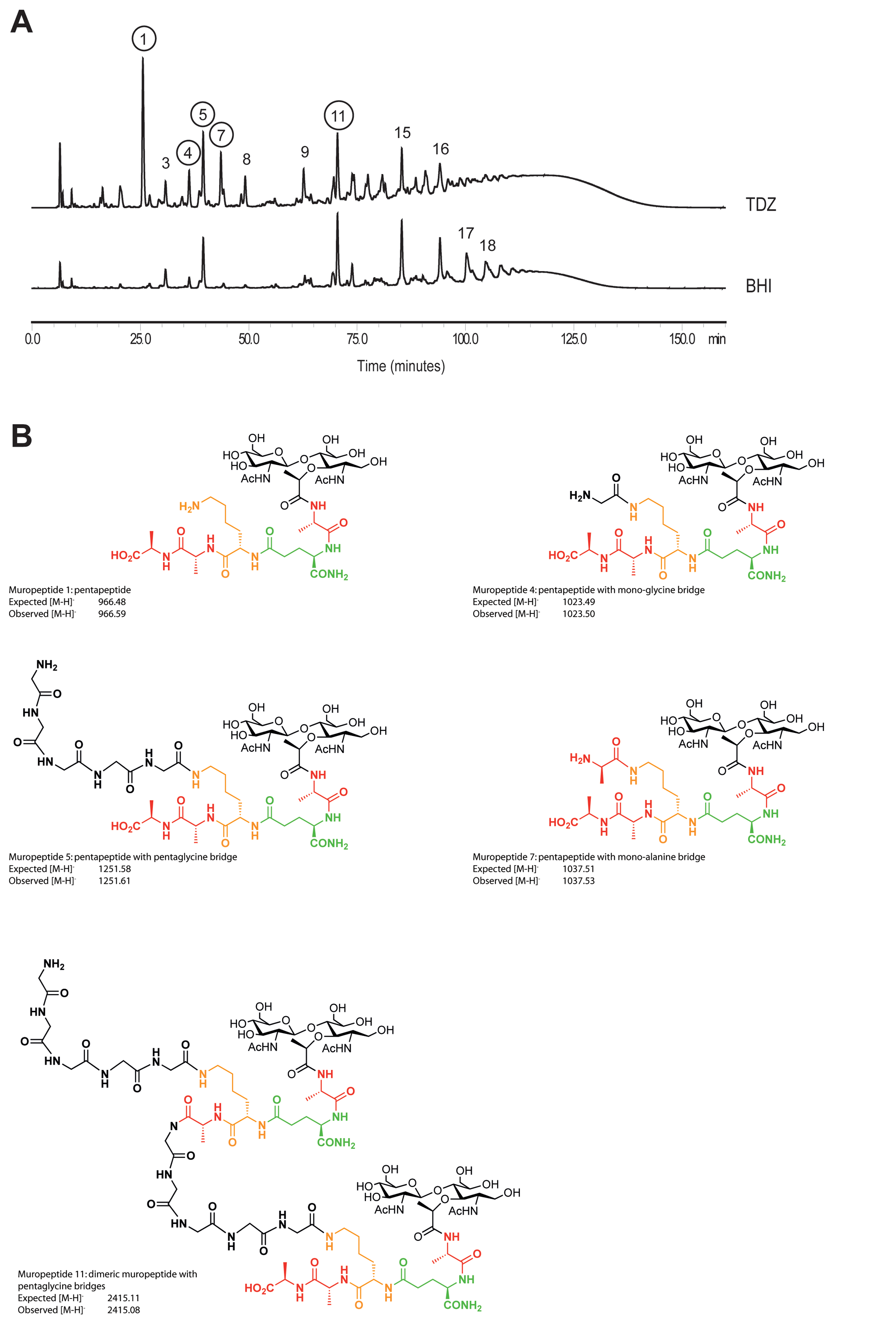

Supplement: Figure S5 — Analysis of the muropeptide composition of the cell wall PGN of S. aureus USA300 strain grown in the presence of TDZ. (A) HPLC profiles of muropeptides released by mutanolysin digestion of the PGN purified from USA300, grown in the presence of TDZ (TDZ) or in its absence (BHI). Peaks were labeled from 1 to 18 according to [30]. In the presence of TDZ, there was accumulation of monomeric muropeptides, lacking the pentaglycine bridge (peak 1) or with an abnormal monoalanine bridge (peak 7). (B) Mass spectrometry analysis of different muropeptide peaks allowed their identification (shown as molecular structures). The expected and the observed masses are also shown. For clarity of the representation, each aminoacid is colored differently (alanine – red, glutamine – green, lysine – orange, glycine – black). (TIF) [file pone.0064518.s005.tif]
