# Supplementary material for: Thioridazine Induces Major Changes in Global Gene Expression and Cell Wall Composition in Methicillin-Resistant Staphylococcus aureus USA300
Source: PLoS One. 2013 May 17;8(5):e64518. doi: 10.1371/journal.pone.0064518 (PMC3656896; doi:10.1371/journal.pone.0064518)
Supplement: Table S1 — Primers used in this study. (PDF) [file pone.0064518.s006.pdf]

**Table S1. Primers used in this study.**

| Gene name | Gene ID | Sequence (5'-3'), forward/reverse primer |
|---|---|---|
| *rpoA* | SAUSA300_2178 | ATGAAGGCGAAGTAACAGCAA/TGCAATTTTAAGCTCTGGGTTT |
| *gyrB* | SAUSA300_0005 | GAAGCATTAGCTGGTTATGCAA/CCACGTCCGTTATCCGTTAC |
| *mecA* | SAUSA300_0032 | ACGGTAACATTGATCGCAAC/AATGACGCTATGATCCCAATC |
| *relA* | SAUSA300_1590 | ACCCGCTCGAAATCCAAATA/GTAAGCCCAGTGTGCTGCAA |
| *saeR* | SAUSA300_0691 | TTGAACAACTGTCGTTTGATGA/ACGCATAGGGACTTCGTGAC |
| *sirA* | SAUSA300_0117 | ATATCAAGGTGCCACTGACG/TTCGGTTTTTGTGTCCATGA |
| *saeP* | SAUSA300_0693 | AATCATCAAAAGGTCCAGATTTAT/TCAACCATTGCGATTTCTTT |
| *cspB* | SAUSA300_2639 | CGCTAGCGATGGCTACAAAA/GGTCTCCACGTTGACCTTCA |
| *coa* | SAUSA300_0224 | TACGGCTGGTAAAGCTGAAGA/CTGTAATTGTGCCCTGTGGA |
| *ctsR* | SAUSA300_0507 | TGGTGGTTACATCCGAATCA/GTCCAATCAGCTGAAGCAAA |
| *sarA* | SAUSA300_0605 | TGGTCACTTATGCTGACAAATTAAA/TGTCAATACAGCGAATTCTTCA |
| *oppB* | SAUSA300_0887 | CAGTAGCTGGATGGGAAGGT/TGGCGACAGTTGCTAAAACA |
| | SAUSA300_0986 | TGGCATTTCGATACATCTTCC/CGCACCTTTAACTTGCTGAGT |
| *ilvD* | SAUSA300_2006 | TTGGTTTGACGGCGTATTTT/TTTGTCCTCATGGCTGCTAA |
| *spsA* | SAUSA300_0867 | GCCAAACCTGGTCAATCAAT/TTGGCATAAGATGCGTCAAC |
| *accB* | SAUSA300_1564 | GCACACAGTAATCAATCAAATCAA/GTCGAAACATCGGAGTTATCA |
| *vraR* | SAUSA300_1865 | GGTAAAGAAGCAATTGCCAAA/CACCATCCATGTCATCCATAA |
