# Supplementary material for: Thioridazine Induces Major Changes in Global Gene Expression and Cell Wall Composition in Methicillin-Resistant Staphylococcus aureus USA300
Source: PLoS One. 2013 May 17;8(5):e64518. doi: 10.1371/journal.pone.0064518 (PMC3656896; doi:10.1371/journal.pone.0064518)
Supplement: Table S2 — Genes upregulated by TDZ exposure. (PDF) [file pone.0064518.s007.pdf]

Table S2. Genes upregulated by TDZ exposure

Functional group and annotation[a]		Gene	Assignment	log_2 expression ratio		Early CWB[c]	D-CYC[d]	VAN[e]	BAC[f]	OXA[g]	Δ*codY*[h]
USA300	N315[b]			TDZ vs. untreated	TDZ + DCX vs. untreated						
**Amino acid biosynthesis***											
SAUSA300_0357	SA0344	*metE*	5-methyltetrahydropteroyltriglutamate--homocysteine S-methyltransferase	1.7	1.9			up			up
SAUSA300_0358	SA0345		putative 5-methyltetrahydrofolate--homocysteine methyltransferase		1.1			up			up
SAUSA300_0359	SA0346		trans-sulfuration enzyme family protein		1.1	up		up			**up**
SAUSA300_0445	SA0430	*gltB*	glutamate synthase, large subunit	1.8	2.0	up				up	**up**
SAUSA300_0446	SA0431	*gltD*	glutamate synthase, small subunit	1.5	1.6	up	up	up	up	up	**up**
SAUSA300_0860	SA0818	*rocD*	Ornithine aminotransferase		1.1	up					
SAUSA300_0864	SA0822	*argG*	argininosuccinate synthase		1.1						
SAUSA300_1225	SA1163		aspartate kinase	3.1	3.3	up		up			**up**
SAUSA300_1226	SA1164	*dhoM*	homoserine dehydrogenase	2.5	2.7	up		up			**up**
SAUSA300_1227	SA1165	*thrC*	threonine synthase	2.7	2.9	up	up	up	up	up	up
SAUSA300_1228	SA1166	*thrB*	homoserine kinase	2.8	2.9	up	up		up	up	up
SAUSA300_1264	SA1201	*trpD*	anthranilate phosphoribosyltransferase		1.1						
SAUSA300_1265	SA1202	*trpC*	indole-3-glycerol phosphate synthase	1.7	2.2						**up**
SAUSA300_1266	SA1203	*trpF*	N-(5phosphoribosyl)anthranilate isomerase		1.4						up
SAUSA300_1267	SA1204	*trpB*	tryptophan synthase, beta subunit	1.1	1.4						up
SAUSA300_1286	SA1225	*lysC*	aspartate kinase	3.7	4.2						**up**
SAUSA300_1287	SA1226	*asd*	aspartate semialdehyde dehydrogenase	4.0	4.5	up		up			**up**
SAUSA300_1288	SA1227	*dapA*	dihydrodipicolinate synthase	3.6	4.0	up		up			up
SAUSA300_1289	SA1228	*dapB*	dihydrodipicolinate reductase	3.0	3.3	up		up	up		up
SAUSA300_1290	SA1229	*dapD*	tetrahydrodipicolinate acetyltransferase	2.8	3.2	up		up	up		up
SAUSA300_1293	SA1232	*lysA*	diaminopimelate decarboxylase	1.3	1.5				up		up
SAUSA300_1299	SA1238		putative tellurite resistance protein	1.5	1.6	up					
SAUSA300_1670	SA1545	*serA*	D-3-phosphoglycerate dehydrogenase	1.8	1.9	up		up	up		up
SAUSA300_2006	SA1858	*ilvD*	dihydroxy-acid dehydratase	1.4	1.7	up	up				**up**
SAUSA300_2007	SA1859	*ilvB*	acetolactate synthase, large subunit	1.7	2.0	up	up				up
SAUSA300_2008	SA1860	*ilvN*	acetolactate synthase, small subunit	2.4	2.8	up	up				up
SAUSA300_2009	SA1861	*ilvC*	ketol-acid reductoisomerase	2.4	2.8	up	up		up	up	**up**
SAUSA300_2010	SA1862	*leuA*	2-isopropylmalate synthase	2.2	2.6	up					up
SAUSA300_2011	SA1863	*leuB*	3-isopropylmalate dehydrogenase	1.8	2.1	up	up				up
SAUSA300_2012	SA1864	*leuC*	3-isopropylmalate dehydratase, large subunit	1.6	2.0	up					**up**
SAUSA300_2013	SA1865	*leuD*	3-isopropylmalate dehydratase, small subunit	1.5	1.8	up					up
SAUSA300_2014	SA1866	*ilvA*	threonine dehydratase	1.5	1.7	up					up
SAUSA300_2605	SA2464	*hisIE*	histidine biosynthesis bifunctional protein hisIE	2.6	2.8			up			up
SAUSA300_2606	SA2465	*hisF*	imidazole glycerol phosphate synthase subunit hisF	2.6	2.9			up			up
SAUSA300_2607	SA2466	*hisA*	phosphoribosylformimino-5-aminoimidazole carboxamide ribotide isomerase hisA	2.7	3.1			up			up
SAUSA300_2608	SA2467	*hisH*	imidazole glycerol phosphate synthase subunit hisH	2.8	3.0		up	up			up
SAUSA300_2609	SA2468	*hisB*	imidazole glycerol phosphate dehydratase hisB	3.3	3.6			up			up
SAUSA300_2610	SA2469	*hisC*	histidinol-phosphate aminotransferase hisC	3.4	3.7						
SAUSA300_2611	SA2470	*hisD*	histidinol dehydrogenase hisD	3.2	3.5		up	up			up
SAUSA300_2612	SA2471	*hisG*	ATP phosphoribosyltransferase hisG	3.1	3.5		up	up			up
**Biosynthesis of cofactors, prosthetic groups, and carriers**											
SAUSA300_1714	SA1588	*ribE*	riboflavin synthase, alpha subunit		1.0			up			
**Cell envelope**											
SAUSA300_0079	÷		putative lipoprotein	1.0	1.0						
SAUSA300_0419	SA0397	*lpl2*	staphylococcus tandem lipoprotein		1.1						up

Table S2 continued…

Functional group and annotation[a]		Gene	Assignment	log_2 expression ratio		Early CWB[c]	D-CYC[d]	VAN[e]	BAC[f]	OXA[g]	Δ*codY*[h]
USA300	N315[b]			TDZ vs. untreated	TDZ + DCX vs. untreated						
SAUSA300_0622	SA0591		putative membrane protein	2.2	1.8		up		up	up	
SAUSA300_0689	SA0659		glycosyl transferase, group 2 family protein		1.0						
SAUSA300_0891	SA0849	*oppA*	oligopeptide ABC transporter, substrate-binding protein	2.8	3.1	up	up	up	up	up	up
SAUSA300_0934	SA0886		membrane protein		1.1	up	up				up
SAUSA300_0953	SA0903		putative membrane protein	1.1			up		up	up	
SAUSA300_1292	SA1231	*alr2*	alanine racemase	2.1	2.5	up					up
SAUSA300_1492	SA1361		putative lipoprotein	1.2	1.2						
SAUSA300_1561	SA1432		putative membrane protein	1.7	1.8		up	up	up	up	
SAUSA300_1761	÷	*epiE*	lantibiotic epidermin immunity protein F		1.0						
SAUSA300_2078	SA1926	*murZ*	UDP-N-acetylglucosamine 1-carboxyvinyltransferase	1.2	1.1		up	**up**	up	up	
SAUSA300_2448	SA2299		putative membrane protein	1.1	1.2		up			up	
SAUSA300_2614	SA2473		putative lipoprotein		1.1	up					up
**Cellular processes**											
SAUSA300_0190	SA0182	*ipdC*	indole-3-pyruvate decarboxylase	1.7	1.9						up
SAUSA300_0959	SA0909	*fmtA*	fmt protein	2.6	2.4		up	**up**	up	up	
SAUSA300_1232	SA1170	*katA*	catalase		1.0	up					up
SAUSA300_1298	SA1237		putative XpaC protein	1.5	1.6					up	
SAUSA300_1844	SA1680	*bcp*	bacterioferritin comigratory protein		1.1						
SAUSA300_2302	SA2146	*tcaA*	teicoplanin resistance associated membrane protein TcaA protein	2.0	1.7		up	**up**	up	up	
SAUSA300_2545	SA2405	*betA*	choline dehydrogenase		1.1		up		up	up	
SAUSA300_2546	SA2406	*betB*	glycine betaine aldehyde dehydrogenase	1.2	1.5						
SAUSA300_2631	SA2490		putative N-acetyltransferase	1.4	1.4	up	up		up	up	
**Central intermediary metabolism**											
SAUSA300_0859	SA0817		NADH-dependent flavin oxidoreductase		1.1						
SAUSA300_1565	SA1436		putative urea amidolyase	3.0	3.1		up	up		up	
SAUSA300_1669	SA1544		aminotransferase, class V	2.4	2.6			up			**up**
SAUSA300_2159	SA2001		aldo/keto reductase family protein	1.4	1.4						
SAUSA300_2497	SA2347		aminotransferase, class I	1.5	1.7	up		up			**up**
SAUSA300_2554	SA2413	*cysJ*	sulfite reductase flavoprotein	1.2	1.0			**up**			
**DNA metabolism**											
pUSA03_0001	÷	*repA*	replication initiator protein		1.1						
SAUSA300_1854	SA1690		regulatory protein RecX	1.4	1.5						
SAUSA300_1876	SA1711		DNA polymerase IV	1.1				**up**			
**Energy metabolism**											
SAUSA300_0055	÷		alcohol dehydrogenase, zinc-containing	2.4	2.7						
SAUSA300_0220	SA0218	*pflB*	formate acetyltransferase	1.1			down		down	down	
SAUSA300_0234	SA0231		putative flavohemoprotein	1.2	1.2		down		down	down	
SAUSA300_0964	SA0914		chitinase-related protein	2.2	2.0		up	**up**	up	up	
SAUSA300_0986	SA0937	*cydA*	cytochrome D ubiquinol oxidase, subunit I	1.7	1.6				up		
SAUSA300_0987	SA0938	*cydB*	cytochrome D ubiquinol oxidase, subunit II	1.5	1.5						
SAUSA300_1246	SA1184	*citB*	aconitate hydratase	1.1	1.2			up		up	
SAUSA300_1640	SA1517	*citC*	isocitrate dehydrogenase, NADP-dependent	1.4	1.6			up		up	
SAUSA300_1641	SA1518	*citZ*	citrate synthase II	1.1	1.3			up		up	
SAUSA300_2463	SA2312	*ddh*	D-lactate dehydrogenase	1.3	1.4						
SAUSA300_2601	SA2461	*icaB*	intercellular adhesion protein B	1.3	1.4						**up**

Table S2 continued…

Functional group and annotation[a]		Gene	Assignment	$\log_2$ expression ratio		Early CWB[c]	D-CYC[d]	VAN[e]	BAC[f]	OXA[g]	Δ*codY*[h]
USA300	N315[b]			TDZ vs. untreated	TDZ + DCX vs. untreated						
**Fatty acid and phospholipid metabolism**											
SAUSA300_1563	SA1434	*accC*	acetyl-CoA carboxylase, biotin carboxylase	2.2	2.4		up	up	up	up	
SAUSA300_1564	SA1435	*accB*	acetyl-CoA carboxylase, biotin carboxyl carrier protein	2.9	3.0			up		up	
SAUSA300_1667	SA1542		putative glycerophosphoryl diester phosphodiesterase	1.1	1.2						
**Hypothetical proteins**											
pUSA03_0030	÷		conserved hypothetical protein	1.6	1.8						
SAUSA300_0081	SA0078		conserved hypothetical protein	1.2	1.4						
SAUSA300_0198	÷		conserved hypothetical protein	1.6	1.8						
SAUSA300_0199	÷		conserved hypothetical protein	1.4	1.6						
SAUSA300_0207	SA0205		conserved hypothetical protein	2.0	2.0						
SAUSA300_0232	SA0230		conserved hypothetical protein	1.1	1.1						
SAUSA300_0356	SA0343		conserved hypothetical protein		1.1			up			up
SAUSA300_0385	SA0372		conserved hypothetical protein		1.1						
SAUSA300_0508	SA0481		conserved hypothetical protein		1.0	up		up			
SAUSA300_0561	SA0535	*vraC*	hypothetical protein	1.8	1.7		up				
SAUSA300_0781	SA0752		conserved hypothetical protein		1.2						
SAUSA300_0866	SA0824		conserved hypothetical protein	1.7	1.5				up		
SAUSA300_0903	SA0860		conserved hypothetical protein	1.1	1.1						
SAUSA300_0929	SA0883		conserved hypothetical protein	3.5	3.7	up					**up**
SAUSA300_0942	SA0892		conserved hypothetical protein	1.3	1.4						
SAUSA300_0957	SA0907		conserved hypothetical protein	1.0							
SAUSA300_1069	SA1018		conserved hypothetical protein	1.0	1.1						
SAUSA300_1107	SA1057		conserved hypothetical protein	1.3	1.4						
SAUSA300_1180	SAS037		conserved hypothetical protein	1.6	1.5						
SAUSA300_1224	SA1162		conserved hypothetical protein	1.2	1.1						
SAUSA300_1277	SA1215		conserved hypothetical protein	1.0			up		up	up	
SAUSA300_1296	SA1235		conserved hypothetical protein	1.5	1.4	up					
SAUSA300_1297	SA1236		conserved hypothetical protein	1.6	1.7	up				up	
SAUSA300_1314	SA1254		conserved hypothetical protein	1.8	1.7	up	up	**up**	up	up	
SAUSA300_1338	SA1280		conserved hypothetical protein		1.1						
SAUSA300_1339	SA1281		conserved hypothetical protein	1.3	1.3						
SAUSA300_1493	SA1362		conserved hypothetical protein	1.1	1.0						
SAUSA300_1566	SA1437		conserved hypothetical protein	2.7	2.8			up		up	
SAUSA300_1606	SA1476		conserved hypothetical protein	2.3	2.2			**up**			
SAUSA300_1671	SA1546		conserved hypothetical protein	1.5	1.6	up		up			**up**
SAUSA300_1723	SA1597		conserved hypothetical protein	1.1							
SAUSA300_1867	SA1702	*yvqF*	conserved hypothetical protein	2.6	2.5		up	**up**	up	up	
SAUSA300_1868	SA1703		conserved hypothetical protein	2.7	2.7		up	**up**	up	up	
SAUSA300_1877	SA1712		conserved hypothetical protein	2.6	2.3		up	**up**	up	up	
SAUSA300_2077	SA1925		conserved hypothetical protein	1.4	1.3					up	
SAUSA300_2261	SA2105		conserved hypothetical protein	1.0	1.0						
SAUSA300_2269	SA2113		conserved hypothetical protein	3.3	2.8			**up**			
SAUSA300_2378	SA2221		conserved hypothetical protein	2.2	2.1	up	up	**up**	up	up	
SAUSA300_2418	SA2262		conserved hypothetical protein	1.1	1.2						
SAUSA300_2421	SA2265		conserved hypothetical protein	1.3	1.4						
SAUSA300_2446	SA2297		conserved hypothetical protein	2.0	2.0		up	**up**	up	up	
SAUSA300_2475	SA2325		conserved hypothetical protein		1.0						
SAUSA300_2493	SA2343	*cwrA*	conserved hypothetical protein	5.1	4.7		up	**up**	up	up	

Table S2 continued…

Functional group and annotation[a]		Gene	Assignment	$\log_2$ expression ratio		Early CWB[c]	D-CYC[d]	VAN[e]	BAC[f]	OXA[g]	Δ*codY*[h]
USA300	N315[b]			TDZ vs. untreated	TDZ + DCX vs. untreated						
SAUSA300_2604	÷		conserved hypothetical protein	2.1	2.6						
SAUSA300_2613	SA2472		conserved hypothetical protein	3.0	3.2						**up**
SAUSA300_2615	SA2474		conserved hypothetical protein	1.5	1.7			up			up
SAUSA300_2618	SA2477		conserved hypothetical protein	2.2	2.4	up					up
SAUSA300_2619	SA2478		conserved hypothetical protein	2.0	2.3	up					up
SAUSA300_2621	SA2480	*drp35*	conserved hypothetical protein	1.1				**up**			
**Mobile and extrachromosomal element functions**											
SAUSA300_1434	÷		phiSLT ORF104a-like protein, repressor	1.2	1.5						
SAUSA300_1435	÷		phiSLT ORF153-like protein	1.0	1.3						
SAUSA300_1436	÷		phiSLT ORF144-like protein, putative lipoprotein		1.1						
SAUSA300_1438	SA1835	*int*	phiSLT ORF401-like protein, integrase	1.0	1.2						
**Protein fate**											
SAUSA300_0867	SA0825	*spsA*	signal peptidase IA	1.4	1.2			**up**			
SAUSA300_0868	SA0826	*spsB*	signal peptidase IB	1.2							
SAUSA300_0877	SA0835	*clpB*	Chaperone clpB	1.3	1.5	up		up		up	
SAUSA300_1278	SA1216	*pepF*	oligoendopeptidase F	1.1	1.1					up	
SAUSA300_1291	SA1230		hippurate hydrolase	2.6	3.0	up		up			up
SAUSA300_1313	SA1253	*ctpA*	carboxyl-terminal protease	1.3	1.3			**up**			
SAUSA300_1316	SA1256	*msrB*	methionine-R-sulfoxide reductase	1.1	1.1	up	up		up	up	
SAUSA300_1317	SA1257	*msrA*	methionine-S-sulfoxide reductase	1.2	1.1	up	up		up	up	
SAUSA300_1654	SA1530	*pepQ*	proline dipeptidase	1.2	1.2						
SAUSA300_1674	SA1549	*htrA*$_1$	putative serine protease HtrA	2.2	2.1			**up**			
SAUSA300_1790	SA1659	*prsA*	foldase protein PrsA precursor	3.0	3.0		up	**up**	up	up	
SAUSA300_1855	SA1691	*sgtB*	monofunctional glycosyltransferase	2.6	2.4	up	up	**up**	up	up	
SAUSA300_2588	SA2446	*secY*	preprotein translocase, SecY protein	1.0	1.1		up				
**Protein synthesis**											
SAUSA300_0736	SA0707	*yfiA*	ribosomal subunit interface protein	3.2	3.5	up		up			up
SAUSA300_1365	SA1308	*rpsA*	30S ribosomal protein S1	1.5	1.6						
SAUSA300_1525	SA1394	*glyS*	glycyl-tRNA synthetase	1.3	1.2						
**Purines, pyrimidines, nucleosides, and nucleotides**											
SAUSA300_0966	SA0916	*purE*	phosphoribosylaminoimidazole carboxylase, catalytic subunit	1.3							
SAUSA300_0967	SA0917	*purK*	phosphoribosylaminoimidazole carboxylase, ATPase subunit	1.1							up
SAUSA300_0968	SA0918	*purC*	phosphoribosylaminoimidazole-succinocarboxamide synthase	2.1	1.7						up
SAUSA300_0969	SA0919	*purS*	phosphoribosylformylglycinamidine synthase	1.8	1.5						up
SAUSA300_0970	SA0920	*purQ*	phosphoribosylformylglycinamidine synthase I	1.9	1.5						up
SAUSA300_0971	SA0921	*purL*	phosphoribosylformylglycinamidine synthase II	2.0	1.6						up
SAUSA300_0972	SA0922	*purF*	amidophosphoribosyltransferase	1.5	1.1						up
SAUSA300_0973	SA0923	*purM*	phosphoribosylformylglycinamidine cyclo-ligase	1.4	1.1						up
SAUSA300_0974	SA0924	*purN*	phosphoribosylglycinamide formyltransferase	1.7	1.3						up
SAUSA300_0975	SA0925	*purH*	bifunctional purine biosynthesis protein	1.2							
SAUSA300_2551	SA2410	*nrdD*	anaerobic ribonucleotide reductase, large subunit	1.1							
**Regulatory functions**											
SAUSA300_0093	÷		transcriptional regulator, LysR family domain protein		1.1						
SAUSA300_0507	SA0480	*ctsR*	transcriptional regulator CtsR	1.1	1.5	up					

Table S2 continued…

Functional group and annotation[a]		Gene	Assignment	log$_2$ expression ratio		Early CWB[c]	D-CYC[d]	VAN[e]	BAC[f]	OXA[g]	Δ*codY*[h]
USA300	N315[b]			TDZ vs. untreated	TDZ + DCX vs. untreated						
SAUSA300_0509	SA0482		ATP guanido phosphotransferase		1.1	up		up			
SAUSA300_0605	SA0573	*sarA*	staphylococcal accessory regulator A	1.4	1.2						
SAUSA300_0653	SA0622		transcriptional regulator, AraC family		1.0						
SAUSA300_1279	SA1217	*phoU*	phosphate transport system regulatory protein PhoU	1.8	1.5				up		
SAUSA300_2160	SA2002		transcriptional regulator, MerR family	1.7	1.8						
SAUSA300_2259	SA2103		putative transcriptional regulator	2.6	2.4		up	**up**	up	up	
SAUSA300_2445	SA2296		transcriptional regulator, MerR family	2.1	1.9			**up**			
**Signal transduction**											
SAUSA300_1865	SA1700	*vraR*	DNA-binding response regulator	2.6	2.6			**up**			
SAUSA300_1866	SA1701	*vraS*	two-component sensor histidine kinase	2.5	2.5		up	**up**	up	up	
**Transport and binding proteins**											
SAUSA300_0231	SA0229		ABC transporter, substrate-binding protein	1.1							
SAUSA300_0306	SA0294	*brnQ*	branched-chain amino acid transport system II carrier protein	1.0	1.1						up
SAUSA300_0558	SA0531	*proP*	putative proline/betaine transporter	1.1				**up**			
SAUSA300_0611	SA0579		putative Na+/H+ antiporter, MnhB component		1.2						
SAUSA300_0612	SA0580		putative Na+/H+ antiporter, MnhC component		1.1						
SAUSA300_0784	SA0753		LysE/YggA family protein	1.0							**up**
SAUSA300_0796	SA0769		ABC transporter, ATP-binding protein	1.1	1.1						**up**
SAUSA300_0797	SA0770		ABC transporter permease protein	1.3	1.4						**up**
SAUSA300_0798	SA0771		ABC transporter, substrate-binding protein		1.0			up			up
SAUSA300_0887	SA0845	*oppB*	oligopeptide ABC transporter, permease protein	2.8	3.0	up		up			**up**
SAUSA300_0888	SA0846	*oppC*	oligopeptide ABC transporter, permease protein	2.7	3.0	up	up	up	up	up	up
SAUSA300_0889	SA0847	*oppD*	oligopeptide ABC transporter, ATP-binding protein	2.5	2.7	up	up	up	up	up	up
SAUSA300_0890	SA0848	*oppF*	oligopeptide ABC transporter, ATP-binding protein	2.7	3.0	up	up	up	up	up	up
SAUSA300_0941	SA0891		putative ferrichrome ABC transporter		1.1						
SAUSA300_0988	SA0939	*trkA*	potassium uptake protein	1.0							
SAUSA300_1280	SA1218	*pstB*	phosphate ABC transporter, ATP-binding protein	2.2	1.9						
SAUSA300_1281	SA1219	*pstA*	phosphate ABC transporter, permease protein PstA	2.3	2.0						
SAUSA300_1282	SA1220	*pstC*	phosphate ABC transporter, permease protein PstC	2.0	1.8						
SAUSA300_1283	SA1221	*pstS*	phosphate ABC transporter, phosphate-binding protein PstS	2.4	2.2						
SAUSA300_1315	SA1255	*crr*	PTS system, glucose-specific IIA component	1.4	1.3	up	up	**up**	up	up	
SAUSA300_1785	SA1654	*ecsB*	putative ABC transporter protein EcsB	1.1	1.1		up		up	up	
SAUSA300_1786	SA1655	*ecsA*	ABC transporter, ATP-binding protein EcsA	1.1	1.2						
SAUSA300_2306	SA2149		ABC transporter, ATP-binding protein	1.1							
SAUSA300_2307	SA2150		ABC transporter, permease protein	1.3							
SAUSA300_2391	SA2235	*opuCc*	glycine betaine/carnitine/choline ABC transporter	1.2	1.1	up					
SAUSA300_2392	SA2236	*opuCb*	glycine betaine/carnitine/choline ABC transporter	1.3	1.3	up					
SAUSA300_2393	SA2237	*opuCa*	glycine betaine/carnitine/choline ABC transporter ATP-binding protein	1.3	1.4	up	up				
SAUSA300_2434	SA2283		transporter protein	1.0	1.2						
SAUSA300_2538	SA2396		amino acid permease family protein	1.3	1.4	up	down	up	down	down	**up**
SAUSA300_2616	SA2475		cobalt transport family protein	1.1	1.3	up		up			up
SAUSA300_2617	SA2476		putative cobalt ABC transporter, ATP-binding protein	1.2	1.4	up		up			up
SAUSA300_2630	SA2489	*nixA*	high-affinity nickel-transporter	1.8	1.8		up		up	up	
**Unknown function**											
SAUSA300_0129	SA0122	*butA*	Acetoin(diacetyl) reductase	1.4	1.5	up	up	up	up	up	**up**

Table S2 continued…

Functional group and annotation[a]		Gene	Assignment	$\log_2$ expression ratio		Early CWB[c]	D-CYC[d]	VAN[e]	BAC[f]	OXA[g]	Δ*codY*[h]
USA300	N315[b]			TDZ vs. untreated	TDZ + DCX vs. untreated						
SAUSA300_0189	SA0181	*entB*	isochorismatase	1.9	2.2						
SAUSA300_0643	SA0612		acetyltransferase, GNAT family	1.1	1.2	up					
SAUSA300_0688	SA0658		oxidoreductase, aldo/keto reductase family	1.1	1.3						
SAUSA300_1070	SA1019		acetyltransferase, GNAT family		1.1	up			up		
SAUSA300_1257	SA1195	*msrR*	peptide methionine sulfoxide reductase regulator MsrR	1.9	1.5		up		up	up	
SAUSA300_1562	SA1433		LamB/YcsF family protein	2.3	2.4			up			up
SAUSA300_1656	SA1532		universal stress protein family	2.0	2.1	up		up	up		**up**
SAUSA300_1668	SA1543		OsmC/Ohr family protein	1.2	1.4						
SAUSA300_2162	SA2004		M23/M37 peptidase domain protein		1.1						
SAUSA300_2496	SA2346		D-isomer specific 2-hydroxyacid dehydrogenase family protein	1.4	1.6	up		up			**up**
SAUSA300_2573	SA2431	*isaB*	immunodominant antigen B		1.2			up			

[a] JCVI categories. Overrepresented categories as determined by Fisher's exact test (Benjamini-Hochberg corrected p-value < 0.05) following TDZ and TDZ + DCX treatment are marked by an asterisk (*).

[b] Based on homology (Biocyc.org).

[c] Comparison to inhibition of early cell wall biosynthesis (CWB) (O'Neill *et al.* (2009). *Antimicrob Agents Chemother* 53: 1701-4). "down" and "up" indicate genes that were commonly down- or upregulated, respectively, in response to inhibition/depletion of MurA/MurZ, MurB, and MurE in *S. aureus*.

[d] "up" indicate genes that were upregulated following treatment of *S. aureus* with D-cycloserine (D-CYC) for 1 hr resulting in 44 % growth inhibition. "down" indicate genes that were commonly downregulated in response to D-CYC, BAC, and OXA (Utaida *et al*. (2003). *Microbiol* 149: 2719-32).

[e] "down" and "up" indicate genes that were down- or upregulated, respectively, following treatment of *S. aureus* with vancomycin (VAN) at 10x MIC for 10 min (Kuroda *et al*. (2003). *Mol Microbiol* 49: 807-21). Genes that lost regulation in a Δ*vraSR* mutant are shown in boldface typing.

[f] "up" indicate genes that were upregulated following treatment of *S. aureus* with bacitracin (BAC) for 1 hr resulting in 77.5 % growth inhibition. "down" indicate genes that were commonly downregulated in response to D-CYC, BAC, and OXA (Utaida *et al*. (2003). *Microbiol* 149: 2719-32).

[g] "up" indicate genes that were upregulated following treatment of *S. aureus* with oxacillin (OXA) for 1 hr resulting in 32 % growth inhibition. "down" indicate genes that were commonly downregulated in response to D-CYC, BAC, and OXA (Utaida *et al*. (2003). *Microbiol* 149: 2719-32).

[h] Differential gene expression in a Δ*codY::ermC* mutant compared to the wild type in exponential growth phase. Genes that were overexpressed in the mutant are indicated by "up", whereas underexpressed genes are indicated by "down". Boldface typing indicates that a region of enrichment was found within the highlighted gene or within the intergenic region upstream of the highlighted gene in a CodY pulldown experiment as described by Majerczyk *et al.* (Majerczyk *et al.* (2010). *J Bacteriol* 192: 2861-77).
