# Supplementary material for: Thioridazine Induces Major Changes in Global Gene Expression and Cell Wall Composition in Methicillin-Resistant Staphylococcus aureus USA300
Source: PLoS One. 2013 May 17;8(5):e64518. doi: 10.1371/journal.pone.0064518 (PMC3656896; doi:10.1371/journal.pone.0064518)
Supplement: Table S3 — Genes downregulated by TDZ exposure. (PDF) [file pone.0064518.s008.pdf]

Table S3. Genes downregulated by TDZ exposure.

Functional group and annotation[a]		Gene	Assignment	log$_2$ expression ratio		Early CWB[c]	CWSS[d]	VAN[e]	Δ*codY*[f]
USA300	N315[b]			TDZ vs. untreated	TDZ + DCX vs. untreated				
**Amino acid biosynthesis**									
SAUSA300_0209	SA0207		putative maltose ABC transporter, maltose-binding protein	-1.3	-1.3				
SAUSA300_0433	SA0418	*cysM*	cysteine synthase/cystathionine beta-synthase	-1.0	-1.1			up	
SAUSA300_0434	SA0419	*metB*	cystathionine gamma-synthase	-1.0	-1.1			up	
SAUSA300_1052	SA1000		fibrinogen-binding protein	-3.4	-3.4				up
SAUSA300_2273	SA2117		Na+/H+ antiporter family protein		-1.0				
SAUSA300_2454	SA2303		membrane spanning protein	-3.4	-3.2		down		
**Biosynthesis of cofactors, prosthetic groups, and carriers**									
SAUSA300_0307	SA0295		5'-nucleotidase, lipoprotein e(P4) family	-1.6	-1.5				up
SAUSA300_0696	SA0666		6-pyruvoyl tetrahydrobiopterin synthase-like protein	-1.0	-1.2			down	
SAUSA300_1661	SA1537	*thiI*	thiamine biosynthesis protein ThiI		-1.0			down	
SAUSA300_1662	SA1538		aminotransferase, class V	-1.1	-1.1			down	
**Cell envelope***									
SAUSA300_0130	SA0123		NAD-dependent epimerase/dehydratase family protein	-1.3	-1.3				
SAUSA300_0131	SA0124		putative bacterial sugar transferase	-1.5	-1.4				
SAUSA300_0132	SA0125		glycosyl transferase, group 1 family protein	-1.1	-1.2				
SAUSA300_0157	SA0149	*cap5F*	capsular polysaccharide biosynthesis protein Cap5F	-1.4	-1.4				up
SAUSA300_0158	SA0150	*cap5G*	capsular polysaccharide biosynthesis protein Cap5G	-1.1	-1.2				up
SAUSA300_0175	SA0167		putative lipoprotein	-2.9	-3.2			up	
SAUSA300_0176	SA0168		ABC transporter, permease protein	-2.6	-2.8			up	
SAUSA300_0222	SA0220		putative membrane protein		-1.2				
SAUSA300_0224	SA0222	*coa*	staphylocoagulase precursor	-2.6	-2.5			up	
SAUSA300_0257	SA0253	*lrgB*	antiholin-like protein LrgB	-2.3	-2.6				
SAUSA300_0273	SA0268		putative membrane protein	-2.0	-1.8	down			up
SAUSA300_0315	SA0304	*nanA*	N-acetylneuraminate lyase subunit	-1.9	-2.2				
SAUSA300_0346	SA0333		putative membrane protein		-1.1				
SAUSA300_0374	SA0360		putative membrane protein	-1.1	-1.1				
SAUSA300_0408	SA0394		putative surface protein	-3.3	-3.3			up	
SAUSA300_0693	SA0663	*saeP*	putative lipoprotein	-2.7	-2.7				up
SAUSA300_0718	SA0688	*sstA*	iron compound ABC transporter, permease		-1.0				
SAUSA300_1055	SA1003	*efb*	fibrinogen-binding protein	-3.1	-3.1				up
SAUSA300_1324	SA1265		putative membrane protein	-1.0	-1.2				
SAUSA300_1327	SA1267	*ebhA*	cell surface protein	-1.2	-1.3				
SAUSA300_1660	SA1536		putative membrane protein		-1.1				
SAUSA300_1809	SA1676		putative membrane protein	-1.3	-1.5				
SAUSA300_1998	SA1850		putative membrane protein	-1.9	-2.0	down		up	
SAUSA300_2256	SA2100		putative N-acetylmuramoyl-L-alanine amidase		-1.1				
SAUSA300_2282	SA2126	*lyrA*	putative membrane protein	-1.1	-1.2			down	
SAUSA300_2287	SA2131		putative membrane protein	-1.4	-1.4				
SAUSA300_2385	SA2229		putative membrane protein	-1.2	-1.5				
SAUSA300_2435	÷		cell wall surface anchor family protein	-1.1	-1.1				
SAUSA300_2436	SA2285	*sasG*	putative cell wall surface anchor family protein	-1.3	-1.2		down		
SAUSA300_2439	SA2288	*galU*	UTP-glucose-1-phosphate uridylyltransferase	-1.0	-1.2				
SAUSA300_2440	SA2291	*fnbB*	fibronectin binding protein B	-3.1	-3.1				down

Table S3 continued…

Functional group and annotation[a]		Gene	Assignment	log_2 expression ratio		Early CWB[c]	CWSS[d]	VAN[e]	Δ*codY*[f]
USA300	N315[b]			TDZ vs. untreated	TDZ + DCX vs. untreated				
SAUSA300_2441	SA2290	*fnbA*	fibronectin binding protein A	-2.7	-2.7				
**Cellular processes**									
SAUSA300_0120	SA0114	*sbnC*	siderophore biosynthesis protein, lucC family	-1.1	-1.1				
SAUSA300_0194	SA0186		sucrose-specific PTS transporter protein	-1.1	-1.2				
SAUSA300_0354	SA0341	*ltrA*	low temperature requirement protein LtrA	-1.4	-1.6				
SAUSA300_0407	SA0393	*set15*	exotoxin	-3.9	-3.9			up	
SAUSA300_0669	SA0638	*bacA*	undecaprenol kinase	-1.3	-1.3		up		
SAUSA300_0773	SA0743		putative staphylocoagulase	-1.6	-1.7				
SAUSA300_0777	SA0747	*cspC*	cold shock protein	-1.2	-1.7				
SAUSA300_1058	SA1007	*hla*	alpha-hemolysin precursor	-1.1	-1.1				up
SAUSA300_1244	SA1182	*mscL*	large-conductance mechanosensitive channel protein	-1.3	-1.5				
SAUSA300_2249	SA2093	*ssaA*	secretory antigen precursor SsaA	-1.0	-1.1	down		down	
SAUSA300_2356	SA2199	*fmhA*	fmhA protein	-1.3	-1.3				
SAUSA300_2364	SA2206	*sbi*	IgG-binding protein SBI	-3.2	-3.2			up	**up**
SAUSA300_2365	SA2207	*hlgA*	gamma-hemolysin component A	-1.3	-1.6			up	
SAUSA300_2366	SA2208	*hlgC*	gamma-hemolysin component C	-1.3	-1.3			up	
SAUSA300_2503	SA2353		secretory antigen precursor SsaA	-1.2	-1.2				
**Central intermediary metabolism**									
SAUSA300_0229	SA0227		putative acyl-CoA transferase FadX	-1.2	-1.2				
SAUSA300_0318	SA0307		N-acetylmannosamine-6-phosphate 2-epimerase	-1.1	-1.1				
SAUSA300_0464	SA0445		methyltransferase	-1.1	-1.2				
SAUSA300_2255	SA2099		monooxygenase family protein		-1.0				
SAUSA300_2468	SA2317		acetyltransferase, GNAT family	-1.1	-1.2				
SAUSA300_2505	SA2355		acetyltransferase, GNAT family	-1.5	-1.5			down	
**DNA metabolism**									
SAUSA300_0776	SA0746	*nuc*	thermonuclease precursor	-2.9	-2.9				up
SAUSA300_1222	SA1160	*nuc*	thermonuclease	-1.1	-1.3	down			
SAUSA300_2037	SA1885		ATP-dependent RNA helicase		-1.0			down	
**Energy metabolism**									
SAUSA300_0119	SA0113	*sbnB*	ornithine cyclodeaminase	-1.5	-1.3				
SAUSA300_0151	SA0143	*adhE*	alcohol dehydrogenase, iron-containing		-1.0				
SAUSA300_0179	SA0171	*fdh*	putative D-isomer specific 2-hydroxyacid dehydrogenase	-1.7	-1.9			up	
SAUSA300_0458	SA0439		Orn/Lys/Arg decarboxylase	-1.0	-1.1				
SAUSA300_0538	SA0511		NAD-dependent epimerase/dehydratase family	-1.6	-1.8				up
SAUSA300_0960	SA0960	*qoxD*	quinol oxidase, subunit IV		-1.1				
SAUSA300_1331	SA1272	*ald*	alanine dehydrogenase	-1.1	-1.0				
SAUSA300_2152	SA1994	*lacD*	tagatose 1,6-diphosphate aldolase	-1.3	-1.5				
SAUSA300_2165	SA2007	*budA*	alpha-acetolactate decarboxylase		-1.2				
SAUSA300_2278	SA2122	*hutU*	urocanate hydratase	-1.4	-1.5				
SAUSA300_2283	SA2127	*rpiA*	ribose-5-phosphate isomerase A		-1.0				
SAUSA300_2285	SA2129	*galM*	aldose 1-epimerase		-1.1				
SAUSA300_2341	SA2183	*narJ*	respiratory nitrate reductase, delta subunit		-1.1		down		

Table S3 continued…

Functional group and annotation[a]				$\log_2$ expression ratio					
USA300	N315[b]	Gene	Assignment	TDZ vs. untreated	TDZ + DCX vs. untreated	Early CWB[c]	CWSS[d]	VAN[e]	Δ*codY*[f]
SAUSA300_2342	SA2184	*narH*	respiratory nitrate reductase, beta subunit	-1.1	-1.1		down		
SAUSA300_2343	SA2185	*narG*	respiratory nitrate reductase, alpha subunit	-2.1	-2.3		down		
**Fatty acid and phospholipid metabolism**									
SAUSA300_0099	SA0091	*plc*	1-phosphatidylinositol phosphodiesterase	-1.4	-1.4				
SAUSA300_0227	SA0225	*fadD*	acyl-CoA dehydrogenase FadD	-2.7	-2.9				
SAUSA300_0228	SA0226	*fadE*	acyl-CoA synthetase FadE	-2.7	-3.0				
SAUSA300_0320	SA0309	*geh*	triacylglycerol lipase precursor	-2.0	-1.9		down		up
**Hypothetical proteins**									
SAUSA300_0007	SA0007		conserved hypothetical protein	-1.4	-1.6				
SAUSA300_0036	SA0054		conserved hypothetical protein	-1.2	-1.4				
SAUSA300_0088	÷		hypothetical protein		-1.1				
SAUSA300_0146	SA0139		conserved hypothetical protein	-1.0	-1.1				
SAUSA300_0168	SA0160		conserved hypothetical protein	-1.2	-1.2				
SAUSA300_0172	SA0164		conserved hypothetical protein		-1.0			up	
SAUSA300_0173	SA0165		conserved hypothetical protein	-2.7	-3.0			up	
SAUSA300_0174	SA0166		conserved hypothetical protein	-2.6	-2.9			up	
SAUSA300_0177	SA0169		conserved hypothetical protein	-2.6	-2.8			up	
SAUSA300_0178	SA0170		conserved hypothetical protein	-1.7	-1.7			up	
SAUSA300_0192	SA0184		conserved hypothetical protein		-1.0				
SAUSA300_0193	SA0185		conserved hypothetical protein	-1.5	-1.7				
SAUSA300_0215	SA0213		conserved hypothetical protein	-4.1	-4.3	down			
SAUSA300_0261	SA0257		conserved hypothetical protein		-1.1				
SAUSA300_0272	SA0267		conserved hypothetical protein	-1.7	-1.6				up
SAUSA300_0274	SA0269		conserved hypothetical protein	-2.6	-2.2	down			**up**
SAUSA300_0463	SA0444		conserved hypothetical protein		-1.1				
SAUSA300_0692	SA0662	*saeQ*	conserved hypothetical protein	-2.5	-2.5				up
SAUSA300_0768	÷		conserved hypothetical protein	-1.1					
SAUSA300_0940	SA0890		conserved hypothetical protein		-1.2				
SAUSA300_1054	SA1002		conserved hypothetical protein	-1.2	-1.2				
SAUSA300_1056	SA1004		conserved hypothetical protein	-2.8	-2.9				
SAUSA300_1240	SA1178		conserved hypothetical protein		-1.0				
SAUSA300_1796	SA1664		conserved hypothetical protein		-1.0			down	
SAUSA300_1797	SA1665		conserved hypothetical protein	-1.4	-1.7	down		down	
SAUSA300_1858	SA1693		conserved hypothetical protein	-1.1	-1.2				
SAUSA300_1919	SA1754		conserved hypothetical protein	-1.4	-1.4				
SAUSA300_1997	SA1849		conserved hypothetical protein	-1.9	-2.2			up	
SAUSA300_2095	SA1944		conserved hypothetical protein		-1.1				
SAUSA300_2481	SA2331		conserved hypothetical protein		-1.1				
SAUSA300_2482	SA2332		conserved hypothetical protein	-1.0	-1.2	down			
SAUSA300_2528	SA2377		conserved hypothetical protein	-1.3	-1.3				up
SAUSA300_2642	SA2497		conserved hypothetical protein		-1.1				
**Mobile and extrachromosomal element functions**									
SAUSA300_1421	÷		phiSLT ORF122-like protein, DNA polymerase	-1.0					

Functional group and annotation[a]		Gene	Assignment	log_2 expression ratio		Early CWB[c]	CWSS[d]	VAN[e]	Δ*codY*[f]
USA300	N315[b]			TDZ vs. untreated	TDZ + DCX vs. untreated				
SAUSA300_1929	SA1765		phi77 ORF004-like protein, putative phage tail component	-1.1	-1.1				down
SAUSA300_1932	÷		conserved hypothetical phage protein	-1.0					
SAUSA300_1933	÷		hypothetical phage protein	-1.1					
SAUSA300_1934	÷		phi77 ORF020-like protein, phage major tail protein	-1.1					
SAUSA300_1937	÷		phi77 ORF045-like protein	-1.0	-1.0				down
SAUSA300_1938	SA1774		phi77 ORF006-like protein, putative capsid protein		-1.0				
SAUSA300_1939	SA1775		phi77 ORF015-like protein, putative protease	-1.1	-1.1				
SAUSA300_1942	SA1778		conserved hypothetical phage protein	-1.0	-1.0				down
SAUSA300_1943	÷		phi77 ORF040-like protein	-1.1	-1.0				down
**Protein fate**									
SAUSA300_0438	SA0423		CHAP domain family	-2.1	-2.3	down	down	down	
SAUSA300_1890	SA1725		staphopain A	-1.8	-1.6				up
SAUSA300_2572	SA2430	*aur*	zinc metalloproteinase aureolysin	-1.5	-1.4	down			
SAUSA300_2639	SA2494	*cspB*	cold shock protein	-1.4	-2.1				
**Protein synthesis**									
SAUSA300_0009	SA0009	*serS*	seryl-tRNA synthetase	-1.2	-1.3	down			
SAUSA300_1134	SA1084	*rplS*	50S ribosomal protein L19		-1.1				
SAUSA300_1545	SA1414	*rpsT*	30S ribosomal protein S20		-1.1				
SAUSA300_2074	SA1922	*rpmE*	50S ribosomal protein L31 type B		-1.2				
**Purines, pyrimidines, nucleosides, and nucleotides**									
SAUSA300_0025	SA0022	*sasH*	5'-nucleotidase family protein	-1.7	-1.7				
SAUSA300_2073	SA1921	*tdk*	thymidine kinase		-1.1	down			
**Regulatory functions**									
SAUSA300_0110	SA0104		transcriptional regulator, GntR family/aminotransferase	-1.1	-1.2				
SAUSA300_0114	SA0108	*sarS*	staphylococcal accessory regulator	-1.3	-1.3				
SAUSA300_0311	SA0299		PfkB family carbohydrate kinase	-1.0	-1.1				
SAUSA300_2218	SA2062	*sarV*	staphylococcal accessory regulator	-1.1	-1.3			up	
SAUSA300_2247	SA2091	*sarY*	staphylococcal accessory regulator	-2.0	-1.7				
SAUSA300_2248	SA2092		transcriptional regulator, AraC family	-2.0	-1.8				
SAUSA300_2279	SA2123		LysR family regulatory protein	-1.1	-1.3				
SAUSA300_2347	SA2189	*nirR*	nitrite reductase transcriptional regulator NirR	-1.7	-1.7		down		
SAUSA300_2367	SA2209	*hlgB*	gamma-hemolysin component B		-1.1			up	
**Signal transduction**									
SAUSA300_0255	SA0251	*lytR*	sensory transduction protein LytR		-1.0				
**Transport and binding proteins***									
SAUSA300_0068	÷		cadmium-exporting ATPase, truncation	-1.9	-2.6				
SAUSA300_0112	SA0106	*lctP*	L-lactate permease	-1.6	-1.6				
SAUSA300_0115	SA0109	*sirC*	iron compound ABC transporter, permease protein SirC	-1.1	-1.1				
SAUSA300_0116	SA0110	*sirB*	iron compound ABC transporter, permease protein SirB	-1.2	-1.2				
SAUSA300_0117	SA0111	*sirA*	iron compound ABC transporter, iron compound-binding protein SirA	-1.4	-1.3	down			

Table S3 continued…

Functional group and annotation[a]		Gene	Assignment	log$_2$ expression ratio		Early CWB[c]	CWSS[d]	VAN[e]	Δ*codY*[f]
USA300	N315[b]			TDZ vs. untreated	TDZ + DCX vs. untreated				
SAUSA300_0142	SA0135	*phnE*	phosphonate ABC transporter, permease protein		-1.1				
SAUSA300_0143	SA0136	*phnE*	phosphonate ABC transporter, permease protein	-1.0	-1.1				up
SAUSA300_0144	SA0137	*phnC*	phosphonate ABC transporter, ATP-binding protein	-1.6	-1.5				
SAUSA300_0145	SA0138		phosphonate ABC transporter, phosphonate-binding protein	-1.1	-1.0				up
SAUSA300_0191	SA0183	*glcA*	PTS system, glucose-specific IIBC component domain protein	-1.2	-1.3	down			
SAUSA300_0200	SA0198	*oppF*	peptide ABC transporter, ATP-binding protein	-1.3	-1.4				
SAUSA300_0208	SA0206	*msmX*	putative maltose ABC transporter, ATP-binding protein	-1.6	-1.6				
SAUSA300_0216	SA0214	*uhpT*	hexose phosphate transport protein	-1.5	-1.7				
SAUSA300_0271	SA0266		ABC transporter, ATP-binding protein	-1.6	-1.6				up
SAUSA300_0313	SA0302		putative nucleoside permease NupC		-1.0				
SAUSA300_0314	SA0303		sodium:solute symporter family protein	-1.6	-1.8				
SAUSA300_0337	SA0325	*glpT*	glycerol-3-phosphate transporter	-1.6	-1.7	down			
SAUSA300_0382	SA0368		sodium:dicarboxylate symporter family protein	-1.2	-1.3				
SAUSA300_0432	SA0417		sodium dependent transporter		-1.2				
SAUSA300_0506	SA0479	*nupC*	pyrimidine nucleoside transport protein	-1.2	-1.5				
SAUSA300_0598	SA0566		putative iron compound ABC transporter, iron compound-binding protein	-1.2	-1.2				
SAUSA300_1807	SA1674		amino acid ABC transporter, ATP-binding protein	-1.0	-1.2		down		
SAUSA300_2207	SA2050		xanthine/uracil permease family protein		-1.0				
SAUSA300_2235	SA2079		iron compound ABC transporter, iron compound-binding protein	-1.3	-1.6				
SAUSA300_2288	SA2132		ABC transporter, ATP-binding protein	-1.5	-1.3				
SAUSA300_2291	SA2135	*gltS*	sodium/glutamate symporter	-1.1	-1.1				
SAUSA300_2324	SA2167	*scrA*	PTS system, sucrose-specific IIBC component	-1.3	-1.4				
SAUSA300_2349	SA2191		formate/nitrite transporter family protein		-1.1				
SAUSA300_2357	SA2200		ABC transporter, ATP-binding protein	-1.1	-1.3				
SAUSA300_2358	SA2201		ABC transporter, permease protein		-1.0				
SAUSA300_2397	SA2241		putative transport protein	-1.2	-1.3			down	
SAUSA300_2449	SA2300		putative transporter	-2.0	-2.1	down			
SAUSA300_2453	SA2302		ABC transporter, ATP-binding protein	-3.7	-3.4				**up**
SAUSA300_2476	SA2326	*ptsG*	phosphotransferase system, glucose-specific IIABC component	-1.5	-1.7				
SAUSA300_2489	SA2339		similar to antibiotic transport-associated protein	-1.1	-1.3				
SAUSA300_2627	SA2486		2-oxoglutarate/malate translocator		-1.1	down			
**Unknown function**									
SAUSA300_0053	÷	*speG*	spermidine N(1)-acetyltransferase		-1.1				
SAUSA300_0089	SA0085		probable tRNA-dihydrouridine synthase	-1.2	-1.3	down		down	
SAUSA300_0206	SA0204		flavodoxin family protein	-1.2	-1.1				
SAUSA300_0256	SA0252	*lrgA*	holin-like protein lrgA	-2.3	-2.6				
SAUSA300_0312	SA0301		indigoidine synthase family protein	-1.1	-1.3				
SAUSA300_0316	SA0305		ROK family protein	-1.2	-1.2				
SAUSA300_0697	SA0667		exsB protein	-1.0	-1.2				
SAUSA300_2457	SA2306		phospholipase/carboxylesterase family protein	-1.1	-1.2				

[a] JCVI categories. Overrepresented categories as determined by Fisher's exact test (Benjamini-Hochberg corrected p-value < 0.05) following TDZ and TDZ + DCX treatment are marked by an asterisk (*).

[b] Based on homology (Biocyc.org).

[c] Comparison to inhibition of early cell wall biosynthesis (CWB) (O'Neill *et al.* (2009). *Antimicrob Agents Chemother* 53: 1701-4). "down" and "up" indicate genes that were commonly down- or upregulated, respectively, in response to inhibition/depletion of MurA/MurZ, MurB, and MurE in *S. aureus*.

[d] Comparison to the cell wall stress stimulon (CWSS) (Utaida *et al*. (2003). *Microbiol* 149: 2719-32). "down" and "up" indicate genes that were commonly down- or upregulated, respectively, in response to treatment of *S. aureus* with inhibitory concentrations of oxacillin (32 % inhibition), bacitracin (77.5 % inhibition), or D-cycloserine (44 % inhibition) for 1 hr.

[e] down" and "up" indicate genes that were down- or upregulated, respectively, following treatment of *S. aureus* with vancomycin (VAN) at 10x MIC for 10 min (Kuroda *et al*. (2003). *Mol Microbiol* 49: 807-21). Genes that lost regulation in a Δ*vraSR* mutant are shown in boldface typing.

[f] Differential gene expression in a Δ*codY::ermC* mutant compared to the wild type in exponential growth phase. Genes that were overexpressed in the mutant are indicated by "up", whereas underexpressed genes are indicated by "down". Boldface typing indicates that a region of enrichment was found within the highlighted gene or within the intergenic region upstream of the highlighted gene in a CodY pulldown experiment as described by Majerczyk *et al*. (Majerczyk *et al.* (2010). *J Bacteriol* 192: 2861-77).
